# Cell wall dynamics during apple development and storage involves hemicellulose modifications and related expressed genes

**DOI:** 10.1186/s12870-016-0887-0

**Published:** 2016-09-15

**Authors:** Emmanuelle Dheilly, Sophie Le Gall, Marie-Charlotte Guillou, Jean-Pierre Renou, Estelle Bonnin, Mathilde Orsel, Marc Lahaye

**Affiliations:** 1 INRA UR 1268 Biopolymères, Interactions, Assemblages, F-44316 Nantes, France; 2 IRHS, INRA, AGROCAMPUS-Ouest, Université d’Angers, SFR 4207 QUASAV, 42 rue Georges Morel, 49071 Beaucouzé cedex, France

**Keywords:** Apple, Fruit development, Cell wall polysaccharides, Hemicelluloses, Transcriptomic analysis, Integrative analysis

## Abstract

**Background:**

Fruit quality depends on a series of biochemical events that modify appearance, flavour and texture throughout fruit development and ripening. Cell wall polysaccharide remodelling largely contributes to the elaboration of fleshy fruit texture. Although several genes and enzymes involved in cell wall polysaccharide biosynthesis and modifications are known, their coordinated activity in these processes is yet to be discovered.

**Results:**

Combined transcriptomic and biochemical analyses allowed the identification of putative enzymes and related annotated members of gene families involved in cell wall polysaccharide composition and structural changes during apple fruit growth and ripening. The early development genes were mainly related to cell wall biosynthesis and degradation with a particular target on hemicelluloses. Fine structural evolutions of galactoglucomannan were strongly correlated with mannan synthase, glucanase (GH9) and β-galactosidase gene expression. In contrast, fewer genes related to pectin metabolism and cell expansion (expansin genes) were observed in ripening fruit combined with expected changes in cell wall polysaccharide composition.

**Conclusions:**

Hemicelluloses undergo major structural changes particularly during early fruit development. The high number of early expressed β-galactosidase genes questions their function on galactosylated structures during fruit development and storage. Their activity and cell wall substrate remains to be identified. Moreover, new insights into the potential role of peroxidases and transporters, along with cell wall metabolism open the way to further studies on concomitant mechanisms involved in cell wall assembly/disassembly during fruit development and storage.

**Electronic supplementary material:**

The online version of this article (doi:10.1186/s12870-016-0887-0) contains supplementary material, which is available to authorized users.

## Background

Apple (*Malus domestica*) fruit development involves a series of biochemical events determinant for qualitative traits, such as appearance, flavour and texture [1]. Fruit growth involves cell divisions and cell expansion resulting from a dynamic interplay between cell turgor pressure, cell wall biosynthesis and remodelling [2]. Apple ripening involves starch conversion to simple sugars, skin colour changes, ethylene production, a respiration burst and flesh softening [3]. Reduction in tissue firmness combines a decrease in cell turgor pressure as well as cell wall polysaccharide remodelling and metabolism [4–6].

Cell walls largely contribute to fruit textural characteristics. In apple, like other fleshy fruit, they are made of pectin, hemicellulose and cellulose, together with some structural proteins [6]. Apple cell wall polysaccharide composition and structure varies with genetics, developmental stages and growth conditions [7, 8]. The relative content of the major cell wall sugars represented by galacturonic acid attributed to pectin, and glucose from cellulose and hemicelluloses increase during apple ripening [9, 10]. Galactose and arabinose content decreases during fruit expansion and further declines during ripening [10–13]. This is due in part to β-galactosidases and α-arabinofuranosidases degradation of the galactan and arabinan side chains of the pectic rhamnogalacturonan I (RGI) [6, 14, 15]. Methyl ester substitutions of the homogalacturonan structural domain of pectins (HG) are partly removed by the action of pectin methylesterases (PME) during apple development [16, 17].

This metabolism of pectin increases cell wall porosity, decreases cell adhesion and affects fruit texture [6, 18]. The loss of RGI galactan and arabinan side chains was associated with softening [12], whereas high content of galactan side chains was associated with firmness [19]. A high arabinofuranosidase activity related to *MdAF3* gene expression was reported in mealy apples [15]. Pectin HG structure and its methyl esterification are also important for apple texture. Down regulation of the *MdPG1* gene coding a polygalacturonase maintains fruit firmness during ripening [20]. In contrast local action of PME (MdPME2) was associated with mealiness development [21].

Unlike pectin, the overall apple hemicellulose composition and molecular weight are not significantly affected during fruit development and ripening [22]. However, their structure and interactions with cellulose are likely remodelled, as observed in the changes of activities and gene expression levels of endo-1,4-β-D-glucanase, xyloglucan endotransglycosylase/hydrolase (XTH) and expansin which are involved in cutting, cutting and pasting and breaking hydrogen bonds between xyloglucan and cellulose [14, 17, 23–27].

In addition to cell wall chemistry and macromolecular interactions, apple texture elaboration involves other complex mechanisms related to tissue organization [28–31] and cellular water partition [8, 20, 29, 32].

As the whole fruit development is involved in texture elaboration [30], we investigated the parallel evolutions of cell wall chemical composition and structure with that of cell wall related gene expression during fruit development and cold storage. The transcriptomic analysis focused on genes annotated for cell wall polysaccharide biosynthesis, remodelling and degrading proteins as well as for structural proteins. Because turgor pressure is involved in fruit development and texture, genes annotated for transporters were also analysed. Gene expression results and correlation analyses between biochemical and transcriptomic profiles highlighted new candidate genes and provided new insights into possible coordinated activities involved in cell wall biosynthesis and metabolism during apple development and storage.

## Results

### Cell wall characterization

The global sugar composition of cell wall prepared as an alcohol insoluble material (AIM) was analysed at each developmental and storage stage (Table 1). As expected, apple fruits accumulated starch during the developmental phases reaching 47.1 % of the AIM dry weight at 110DAF. Starch content decreased at harvest and during the cold storage period. The cell wall polysaccharides after deduction of starch glucose content in AIM sugars (non-starch polysaccharides, NSP) were mainly glucose, uronic acids (UA), arabinose and galactose in decreasing order of proportion. The total amount of these 4 main sugars reached 85 to 88 % of NSP depending on developmental and storage stages. Galactose content decreased constantly from 18.7 to 7.2 % of NSP while uronic acids content increased slightly from 22.4 to 29 % of NSP when fruits reached late development stages. Smaller amounts of xylose, mannose, and traces of rhamnose and fucose were also detected. Xylose and fucose contents increased slightly at ripening stages while mannose contents decreased. Acetyl ester content also decreased during the ripening stages from 1.5 % at 60DAF to 1.2 % of NSP at 2 M. In contrast, methyl ester content did not show any significant change.

**Table 1:** Chemical composition of fruit cell wall

Sample	Starch		NSP		Sugar																Acetyl ester		Methyl ester		DM	
					Rha		Fuc		Ara		Xyl		Man		Gal		Glc		UA							
	*		*				*				*		*		*				*		*					
60DAF	18.0	±2.7	69.2	±4.0	1.3	±0.1	0.8	±0.1	16.7	±1.6	4.8	±0.4	4.4	±0.4	18.7	±1.6	31.0	±5.4	22.4	±1.3	1.5	±0.1	2.8	±0.3	69.2	±17.5
110DAF	47.1	±2.2	51.5	±2.4	1.3	±0.1	0.8	±0.1	13.6	±1.3	5.2	±0.4	3.8	±0.3	17.2	±1.7	34.1	±5.6	23.9	±1.7	1.8	±0.3	3.1	±0.2	70.7	±10.4
H	13.4	±2.4	82.3	±3.3	1.4	±0.1	1.2	±0.1	14.6	±0.9	6.9	±0.4	3.3	±0.2	11.3	±0.8	35.3	±3.9	26.1	±1.7	1.4	±0.1	2.8	±0.4	59.4	±16.6
1 M	3.8	±0.9	87.4	±1.9	1.3	±0.0	1.2	±0.0	14.7	±0.3	7.2	±0.2	3.3	±0.1	8.8	±0.7	35.7	±1.1	27.8	±0.7	1.3	±0.1	3.4	±0.1	67.0	±4.8
2 M	1.3	±0.8	90.2	±1.8	1.2	±0.0	1.2	±0.0	14.9	±0.2	7.6	±0.2	3.1	±0.1	7.2	±0.4	35.8	±0.7	29.0	±0.7	1.2	±0.0	2.7	±0.2	52.2	±8.2

Analyses were carried out at 5 time points (60 and 110DAF, H: harvest, 1 M and 2 M: i.e.,1 and 2 months of cold storage). Starch and non-starch polysaccharides (NSP) are expressed as a percentage of AIM dry weight (alcohol insoluble material). Sugars, acetyl and methyl ester contents are expressed as a percentage of NSP. DM: degree of methyesterification. *: significant differences between 60 DAF and 2 M with *p* < 0.0001

### Determination of hemicellulose fine structure

A structural profiling approach by enzymatic digestion coupled with MALDI-TOF MS analysis of the degradation products was used to follow modifications of hemicellulose fine structure. As apple hemicelluloses include xyloglucan (XyG), galactoclucomannan (GgM) and glucuronoarabinoxylan (GAX) [33–35], mannanase, xylanase and glucanase degradations were done sequentially. The order of the enzymatic treatments was chosen to maximise oligosaccharides release.

To facilitate enzymatic treatments, AIM was first washed with water (water soluble fraction, WS, Table 2) and then partially depectinated by pectin lyase and rhamnogalacturonase (pectinase-soluble fraction, PS). Uronic acids (UA) and neutral sugars (NS) contents were analysed after each treatment. In the WS fraction, UA content increased continuously from 0.5 % at 60DAF to 1.3 % of NSP at 2 M (Table 2). In contrast, NS content decreased from 1.3 % at 60DAF to 0.9 % of NSP after 2 months of cold storage (2 M). This decrease affected the mannose, galactose and glucose content, but not that of arabinose, which was the major sugar of this fraction (Table 3A). As expected, the subsequent pectinase treatment (PS) had a drastic effect and removed 15.2 to 25.3 % of NSP depending on the fruit stages (Table 2). The amount of NS content decreased from 20.4 to 10.9 % of NSP from 60DAF to 2 M (Table 2), including the majority of the released rhamnose, arabinose, mannose and galactose (Table 3B). The difference between 60DAF and 2 M was mainly due to the decrease in galactose content in the PS fraction (Table 3A). No significant change was observed in UA content between 60DAF and 2 M (Table 2) but the majority of UA was released with this treatment (Table 3B).

**Table 2:** Soluble acidic and neutral sugars content released by sequential treatment of AIM

Treatment		60DAF		110DAF		H		1 M		2 M		Stat
WS	NSP	1.8	±0.1	2.7	±0.2	2.2	±0.1	1.8	±0.2	2.2	±0.2	c
	UA	0.5	±0.1	1.2	±0.1	0.9	±0.2	0.9	±0.1	1.3	±0.1	abc
	NS	1.3	±0.2	1.4	±0.2	1.3	±0.2	0.9	±0.1	0.9	±0.2	bc
PS	NSP	25.3	±0.6	27.3	±2.7	19.3	±1.2	16.4	±1.3	15.2	±0.9	abc
	UA	5.0	±0.6	5.3	±1.1	4.9	±0.5	4.7	±0.5	4.3	±0.2	
	NS	20.4	±1.8	22.0	±6.2	14.4	±0.9	11.7	±1.3	10.9	±0.9	abc
Mannanase	NSP	2.8	±0.4	3.3	±0.4	1.6	±0.3	1.3	±0.1	1.3	±0.1	ac
	UA	0.0		0.0		0.1	±0.1	0.1	±0.0	0.2	±0.0	ac
	NS	2.8	±0.4	3.3	±0.4	1.5	±0.4	1.2	±0.1	1.1	±0.1	ac
Xylanase	NSP	1.0	±0.2	1.2	±0.4	0.9	±0.2	0.7	±0.1	0.5	±0.1	bc
	UA	0.0		0.0		0.0		0.0		0.0		
	NS	1.0	±0.2	1.2	±0.4	0.9	±0.2	0.7	±0.1	0.5	±0.1	bc
Glucanase	NSP	13.9	±2.2	15.6	±4.2	15.9	±1.4	14.4	±1.9	13.9	±1.8	
	UA	0.0		0.0		0.2	±0.1	0.2	±0.1	0.2	±0.1	ac
	NS	13.9	±2.2	15.6	±4.2	15.7	±2.6	14.2	±2.3	13.7	±2.0	

Analyses were carried out by colorimetric analyses at 5 time points (60 and 110DAF, H: harvest, 1 M and 2 M: i.e.,1 and 2 months of cold storage) on samples released after sequential treatments with water (water-soluble, WS), pectinases (pectinase-soluble, PS), mannanases, xylanase and finally glucanase. Non-starch polysaccharides content (NSP) are expressed as a percentage of AIM dry weight, uronic acids (UA) and neutral sugars (NS) contents are expressed as a percentage of initial NSP dry weight. a, b, c: significant differences between 60 DAF and H, H and 2 M, 60 DAF and 2 M with *p* < 0.0001

**Table 3:** Neutral sugar composition of the fractions released by sequential treatment of AIM

	Treatment	A	% NSP								B	% initial sugar content in NSP							
			NS									NS							
			Rha	Fuc	Ara	Xyl	Man	Gal	Glc	UA		Rha	Fuc	Ara	Xyl	Man	Gal	Glc	UA
60DAF	WS		0.0	0.0	0.2	0.0	0.1	0.2	0.2	0.5		1.3	0.8	1.1	0.4	1.9	1.1	0.7	2.2
	PS		**0.2**	0.0	**1.9**	0.1	**1.0**	**2.1**	1.1	**5.0**		**15.2**	2.1	**11.2**	1.1	**22.7**	**11.2**	3.7	**22.3**
	Mannanase		0.0	0.0	0.3	0.0	0.3	0.3	0.4	0.0		2.6	0.0	1.8	0.9	6.2	1.3	1.2	0.0
	Xylanase		0.0	0.0	0.2	0.0	0.0	0.1	0.1	0.0		1.3	0.0	1.0	0.9	0.1	0.6	0.3	0.0
	Glucanase		0.1	0.2	0.4	**0.6**	0.3	0.3	**5.1**	0.0		5.7	**18.4**	2.1	**13.1**	7.5	1.8	**16.4**	0.0
2 M	WS		0.0	**0.0**	0.2	0.0	0.0	0.1	0.1	1.3		1.9	0.0	1.6	0.4	1.4	2.0	0.2	4.5
	PS		**0.3**	0.0	**2.3**	0.1	**0.9**	**0.6**	0.2	**4.3**		**21.1**	1.5	**15.3**	1.4	**27.8**	**8.8**	0.5	**14.8**
	Mannanase		0.0	0.0	0.2	0.1	0.2	0.1	0.1	0.2		2.0	1.4	1.1	0.8	5.4	1.2	0.3	0.7
	Xylanase		0.1	0.0	0.9	0.5	0.0	0.3	0.2	0.0		5.4	0.0	6.2	6.0	0.0	3.6	0.6	0.0
	Glucanase		0.1	**0.2**	0.4	**1.0**	0.3	0.5	**5.7**	0.2		9.1	**19.8**	2.7	**12.9**	8.2	6.6	**15.8**	0.7

Analyses were carried out at 2 time points (60DAF and 2 M: i.e., 2 months of cold storage) on samples released after sequential treatments with water (water-soluble, WS), pectinases (pectinase-soluble, PS), mannanases, xylanase and finally glucanase. The neutral sugars (NS) were measured by GC and the uronic acids (UA) by colorimetry. The results are expressed A) as percentage of initial NSP in the AIM dry weight (% NSP) and B) as a percentage of the initial amount of each sugar in the NSP fraction of AIM. Numbers in bold are the maximum of released sugars among all treatments

*Rha*, rhamnose, *Fuc*, fucose, *Ara*, arabinose, *Xyl*, xylose, *Man*, mannose, *Gal*, galactose, *Glc*, glucose, *UA*, uronic acids

### Structure of mannose-containing polysaccharides

Endo-β-mannanase treatment on the remaining extracts allowed access to mannan-rich hemicelluloses. The treatment released 2.8 to 3.3 % of NSP at 60DAF and 110DAF and significantly less from harvest to 2 M with only 1.3%NSP (Table 2). No UA was detectable at early stages of development and only traces afterwards (Table 2). At 60DAF, hydrolysis products were mainly composed of glucose, arabinose, mannose and galactose, each representing 0.3 to 0.4 % of NSP (Table 3A) and respectively only 1.2, 1.8, 6.2 and 1.3 % of their respective initial content in NSP, as most of them was already removed in the PS fraction (Table 3B). The lower NS content of the mannanase fraction at 2 M was mainly due to the decline of galactose and glucose content with only 0.1 % NSP remaining for each.

**Fig. 1:**
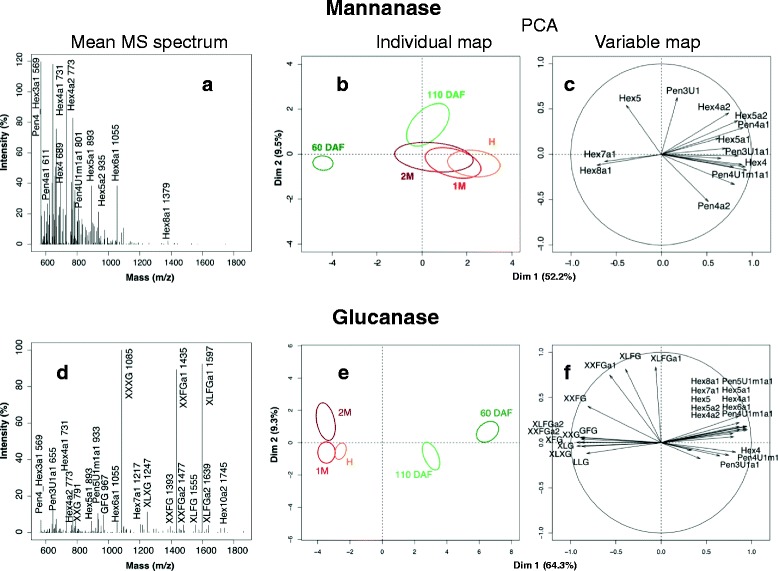
Mean MALDI-TOF MS spectra (**a**, **d**) and principal component analysis (**b**, **c**, **e**, **f**) of MS ions of annotated oligomers in the mannanase (**a**, **b**, **c**) and glucanase (**d**, **e**, **f**) digests. 60 DAF: 60 days after flowering, 110 DAF: 110 days after flowering, H: harvest, 1 M: 1 month of cold storage, 2 M: 2 months of cold storage. Nomenclature of xyloglucan oligosaccharides followed that of [131, 133] extended to account for acetyl groups noted a. In brief, it uses uppercase letters representing an individual 1 → 4 linked β -D-glucose residue and its pendant side chains. Accordingly, bare glucose residue is designated by the letter G while when branched by α -D-xylosyl residue on O-6, it is refers to X. With further extension of the branch by one β-D-galactosyl linked on xylose O-2, the trisaccharide structure formed is referred to L and when the latter is further extended by one α-L-fucose residue linked at O-2 of galactose, the structure is then referred to F. Hexose containing oligosaccharides attributed to galactoglucomannans were noted Hex. Pentose based oligosaccharides were noted Pen. These codes are extended by U, m and a when the residues are substituted by an uronic acid, a methyl and and acetyl group, respectively. The number following the structure codes denoted the number of building structures and substituent groups in the oligosaccharides (i.e., Hex3a2 corresponds to 3 hexoses and 2 acetyl groups; Pen5U1m1a1 corresponds to 5 pentose, 1 uronic acid, 1 methyl and 1 acetyl groups)

Galactoglucomannan fine structures recovered in the mannanase hydrolysates were assessed qualitatively by MALDI-TOF MS spectra analysis (Fig. 1a). As expected, MS spectra showed a series of more or less acetyl-esterified hexo-oligosaccharides with degrees of polymerization from 4 to 8 attributed to mannans/glucomannans/galactoglucomannans fragments. Major fragments in the mean spectrum were attributed to Hex4a1 (4 hexose residues substituted by 1 acetyl group, see legend of Fig. 1 for nomenclature, *m/z* 731), Hex4a2 (*m/z* 773), Hex5a1 (*m/z* 893) Hex5a2 (*m/z* 935), and Hex6a1 (*m/z* 1055) oligomers. An ion with mass corresponding to hexose and pentose containing structures, Hex3a1 and Pen4, respectively, was observed at *mz* 569 (Pen4: 4 pentose residues). Minor structures identified were Hex4 (*m/z* 689), Hex5 (*m/z* 851), Hex5a2 (*m/z* 935), Hex7a1 (*m/z* 1217), Hex7a2 (*m/z* 1259), Hex8a1 (*m/z* 1379) and Hex8a2 (*m/z* 1421). The spectra also revealed the presence of minor pento-oligosaccharides: Pen3U1 (3 pentose residues substituted by 1 uronic acid, *m/z* 613), Pen3U1a1 (*m/z* 655), Pen4a1 (*m/z* 611), Pen4a2 (*m/z* 653), Pen4U1m1 (*m/z* 759), Pen4U1m1a1 (*m/z* 801) and Pen5a1 (*m/z* 743) arising from the minor contamination of the commercial mannanase by xylanase. Principal components analysis (PCA) of annotated oligosaccharides ion intensity was done to provide a synthetic view of sample variations as well as of the variables contributing to these variations. This analysis revealed a clear change in fine structure of mannose-containing hemicelluloses during fruit development particularly during the early phases (Fig. 1b, c). PCA of MS spectra showed that the acetylated oligomers Hex7a1 and Hex8a1 differentiated the fruits at 60DAF.

### Structure of xylose-containing polysaccharides

Treatment of the endo-β-mannanase residues by endo-β-xylanase was performed to analyse xylose-containing hemicelluloses. This treatment released a small amount of neutral sugars, only 1 % of NSP at 60DAF, which decreased during cold storage to 0.5 % of NSP at 2 M (Table 2). No acidic sugar was detected in this fraction (Table 2). Arabinose, galactose and glucose were the main neutral sugars detected at 60DAF with 0.1 to 0.2 % of the initial NSP (Table 3A). After 2 months cold storage, more arabinose (0.9%NSP), galactose (0.3%NSP) and glucose (0.2%NSP) were detected (Table 3A). They represented 6.2, 3.6 and 0.6 % of their respective initial content in NSP (Table 3B). Rhamnose and xylose contents increased in 2 M samples with respectively 0.1 and 0.5 % of the initial NSP, representing 5.4 and 6.0 % of their initial content in NSP. In contradiction with the global NS measurements by colorimetry (Table 2), the GC method showed an increase in neutral sugars released by the treatment after 2 M when compared to 60DAF (Table 3A). Due to the overall low amounts of the xylose-containing oligosaccharides in the hydrolysis products (Table 3B), xylanase hydrolysates were not further analysed.

### Structure of glucose-containing polysaccharides

Endo-β-glucanase was applied on xylanase residues as the last enzymatic treatment to access xyloglucan structures. The treatment solubilized from 13.9 %NSP at 60DAF to a maximum of 15.7 %NSP at harvest stage and decreased to 13.9 % NSP at 2 M (Table 2). Only a very small amount of UA (0.2 % NSP) was released from the harvest stage and thereafter. As expected, the main soluble sugar was glucose with respectively 5.1 and 5.7 % NSP at 60DAF and 2 M (Table 3A) and representing 16.4 % at 60 DAF and 15.8 % at 2 M of the initial content in NSP. With no remarkable differences between 60DAF and 2 M stages, smaller amounts of the other sugars were also solubilized. Most of the released fucose, glucose and xylose were found in the glucanase hydrolysis products (Table 3B).

In consistence with the high content of glucose in the hydrolysates (Table 3), xyloglucan oligosaccharides (XyGOs) were identified by MALDI-TOF MS analysis (Fig. 1b). The mean spectrum revealed the presence of major acetyl-esterified XyGOs: XXFGa1 (*m/z* 1435) and XLFGa1 (*m/z* 1597) together with other structures attributed according to their respective mass to XXG (*m/z* 791), XLG (*m/z* 953), GFG (*m/z* 967), XFG (*m/z* 1099), XLXG (*m/z* 1247), XLXGa1 (*m/z* 1289), XXFG (*m/z* 1393), XLGa1 (*m/z* 1451), XXFGa2 (*m/z* 1477) and XLFGa2 (*m/z* 1639). Minor fragments were also detected as hexo or pento-oligosaccharides and attributed to Hex4 (*m/z* 689), Hex4a1 (*m/z* 731), Hex4a2 (*m/z* 773), Hex5 (*m/z* 851), Hex5a1 (*m/z* 893), Hex5a2 (*m/z* 935), Hex6a1 (*m/z* 1055), Hex7a1 (*m/z* 1217) and Hex8a1 (*m/z* 1379), Pen3U1 (*m/z* 613), Pen3U1a1 (*m/z* 655), Pen4a1 (*m/z* 611), Pen4U1m1 (*m/z* 759), Pen4U1m1a1 (*m/z* 801), Pen5a1 (*m/z* 743) and Pen5U1m1a1 (*m/z* 933). These fragments reflected the activity of the commercial glucananase on glucomannan as well as the presence of minor contaminating xylanolytic activities. If no significant change in global NS composition was observed, a clear change of the oligosaccharide fine structures occurred during fruit development, particularly between the early developmental phases (60DAF and 110DAF) and the matures stages (H, 1 M and 2 M). While most of the XyGOs oligomers, and particularly XXG, GFG, XLXG, XXFGa2, XLFGa2, were representative of mature stages, the hexo and pento-polysaccharides distinguished the spectra of fruits in early development. Particularly, Hex6a1, Hex5a1, Hex7a1, Hex4a1, Hex4a2, Hex5a2, Pen5U1m1a1, Hex8a1, Hex4, Pen4U1m1a1, Hex5, Pen4U1m1 and Pen3U1a1 were representative of the early stages (Fig. 1b).

### Transcriptome profiling

**Fig. 2:**
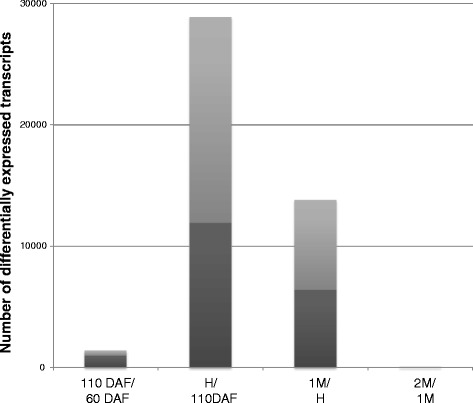
Differentially expressed transcripts during kinetic of apple development and ripening. Graph represents the number of significant differentially expressed transcripts between 2 time points. Transcripts are down-regulated or up-regulated, respectively in dark and in light grey, in comparison with the earlier stage of development. 60 DAF: 60 days after flowering, 110 DAF: 110 days after flowering, H: harvest, 1 M: 1 month of cold storage, 2 M: 2 months of cold storage

In order to identify genes potentially involved in the structural modifications of cell wall polysaccharides, transcriptome analyses were performed on the same samples used for cell wall biochemical analyses. Transcriptomic profiling performed with the AryANE_v1 microarray revealed that 42 % of the tested transcripts were expressed at one or more developmental stages for at least one of the 8 genotypes analysed. Differentially expressed transcripts between subsequent developmental stages were identified with significant *P*-values for t-tests (*P*-value <0.01; Fig. 2). The highest numbers of differentially expressed transcripts were observed between 110DAF and harvest, and harvest and 1 M storage. To study the changes between apple development and fruit ripening, the transcriptome at 60DAF was compared with the transcriptome at 2 M. A total of 23,001 differentially expressed transcripts were identified. Subsequent hierarchical clustering analysis on expression profiles led to the selection of 5150 transcripts displaying similar expression profiles for the 8 genotypes grown in both orchards when considering the 16 time series (Additional file 1). Microarray data were validated by RT-qPCR experiments on a subset of differentially expressed genes, using cDNA from 60DAF and 2 M apple fruits. A similar difference between gene expression levels was observed with both techniques (Pearson correlation = 0.82, *P*-value < 0.01) (Additional file 2).

In AryANE_v1 microarray, sense (S) and antisense (AS) probes were designed for each annotated apple coding DNA sequence (CDS), and 26 % of the differentially expressed probes corresponded to AS transcripts. Celton et al. [36] demonstrated that these AS transcripts were likely to be involved in small interfering RNA (siRNA) dependent negative regulation of the coding mRNAs. This study only considered genes with differentially expressed sense transcripts.

Sense transcripts with higher expression during early fruit development (60DAF and 110DAF: cluster A) or during fruit ripening and cold storage (harvest, 1 M, 2 M: cluster B) were selected for further analyses, and represented respectively 6.2 and 10.5 % of the selected differentially expressed transcripts. Based on genes annotations, they were classified into functional categories. 0.5 % had annotations related to cell wall biosynthesis and/or remodelling, or solutes flux changes (Additional file 3). In order to refine the selection, deduced protein sequence from these genes were analysed for subcellular targeting and protein domain annotation. The potential cellular locations of 96.5 % of the proteins corresponding to these genes were analysed with the ProtAnnDB tool [37] (Additional file 3). In concordance with a function on cell wall modifications, 66 proteins with a signal peptide for endoplasmic reticulum (ER) targeting were potential candidates to be exported to the apoplast. For 21 predicted transporters, 2 had surprisingly no predicted transmembrane domain. 5 of the 6 peroxidases had a predicted signal peptide for ER targeting and 4 were predicted to belong to Class III peroxidase superfamily when analysed through the PeroxiBase tool. Among the enzymes and proteins related to cell wall modification, 15 % could not be allocated to a coherent subcellular compartment. This was probably due to the prediction models used and/or to potentially truncated protein sequences which were deduced from the apple genome sequence and annotation [38]. In addition, some annotations were different between ProtAnnDB and CAZy databases. For example, several proteins were identified as pectin lyase-like with ProtAnnDB, but were grouped as glycoside hydrolases 28 (GH28) (MDP0000147794; MDP0000175027; MDP0000251956; MDP0000270685; MDP0000665344; MDP0000818931 and MDP0000249285) or carbohydrate esterases 8 (CE8) (MDP0000177299; MDP0000212502; MDP0000251256; MDP0000252508; MDP0000287234) in CAZy database. Such discordances probably resulted from the markedly different methods and criteria used for protein annotation.

A total of 114 cell wall related genes were selected, 82 % were expressed during the early developmental phases and the remaining 18 % were expressed during later developmental stages and storage. According to their expression pattern, early expressed genes were grouped in cluster A while the later expressed genes were in cluster B (Table 4; Additional file 3). Cell wall genes from cluster A included mainly genes potentially involved in pectin and cellulose/hemicellulose metabolism, respectively 20 and 26 genes. Several genes potentially coding expansins, galactosyltransferases, glycoproteins and many β-galactosidases were also identified in this cluster, as well as peroxidases and transporters. In contrast, few cell wall related genes were identified in cluster B. Those identified were mainly involved in pectin degradation, and very few genes were involved in cellulose/hemicelluloses metabolism including genes coding expansins (Table 4; Additional file 3).

**Table 4:** Molecular and biochemical function of selected genes potentially involved in cell wall dynamic

Molecular function	Biochemical annotation	Gene_id	
		Cluster A	Cluster B
Pectin biosynthesis	Galacturonosyltransferase (GAUT)	MDP0000179747	MDP0000609623
	Galacturonosyltransferase-like (GATL)	MDP0000124674, MDP0000518347, MDP0000678218, MDP0000794936, MDP0000856834, MDP0000370712	
Pectin degradation	Glycoside hydrolase family 79 (GH79)	MDP0000199066	
	Pectate lyase	MDP0000266603, MDP0000277149, MDP0000319156, MDP0000631698, MDP0000232225, MDP0000394944, MDP0000693765, MDP0000818931	
	Pectin acetylesterase	MDP0000193151, MDP0000834641	
	Pectin esterase	MDP0000177299, MDP0000212502	MDP0000251256, MDP0000252508, MDP0000287234
	Pectin methylesterase inhibitor	MDP0000250584	MDP0000836165
	Polygalacturonase (PG)	MDP0000147794, MDP0000175027, MDP0000251956, MDP0000665344, MDP0000270685	MDP0000249285
Cellulose/Hemicelluloses biosynthesis	UDP-xylosyltransferase	MDP0000197595	
	Cellulose synthase	MDP0000185368, MDP0000322053	
	Cellulose synthase-like A (CSLA)	MDP0000263736, MDP0000133719, MDP0000717000, MDP0000131947, MDP0000659120, MDP0000673496	
	Cellulose synthase-like E (CSLE)		MDP0000196876
Cellulose/Hemicelluloses degradation	α-arabinofuranosidase/α-xylosidase	MDP0000208161	
	α-L-fucosidase	MDP0000166406	MDP0000543167
	β -glucosidase	MDP0000140817	
	Glycoside hydrolase family 1 (GH1)	MDP0000217844, MDP0000147765	
	Glycosyl hydrolase family 9 (GH9)	MDP0000147635, MDP0000131397, MDP0000561662	
	Xyloglucan endotransglycosylase/hydrolase (XTH)	MDP0000180043, MDP0000132431, MDP0000378203	
Glycoproteins	Arabinogalactan protein (AGP)	MDP0000221961, MDP0000893240	
	Fasciclin-like arabinogalactan-protein (FLA)	MDP0000525641, MDP0000658332	
	Hydroxyproline-rich glycoprotein family protein (HRGP)	MDP0000144792, MDP0000697140, MDP0000849284	
	Wall associated kinase (WAK)	MDP0000630155	
	Wall associated kinase-like (WAKL)		MDP0000278145, MDP0000426154
Expansins	Expansin A (EXPA)	MDP0000259640, MDP0000785413, MDP0000257797	
	Expansin-like A (EXLA)	MDP0000568045	MDP0000906812
	Expansin-like B (EXLB)		MDP0000214811, MDP0000292477
Galactosyltransferases	Galactosyltransferase	MDP0000198402, MDP0000237443	
β-galactosidases	β-galactosidases	MDP0000030527, MDP0000195063, MDP0000201058, MDP0000227393, MDP0000310582, MDP0000899966, MDP0000151981, MDP0000265046, MDP0000271897, MDP0000895533, MDP0000682327	MDP0000127542, MDP0000416548, MDP0000944874
Peroxidases	Peroxidase	MDP0000272643, MDP0000678562, MDP0000488361, MDP0000221335, MDP0000122663	MDP0000142485
Transport	H(+)-ATPase	MDP0000810883	
	Anion transporter	MDP0000142911, MDP0000877937	
	K+ transporter	MDP0000414314, MDP0000800190, MDP0000889811, MDP0000170687, MDP0000853168, MDP0000778372	MDP0000403872
	Cation transporter	MDP0000470237	
	Zinc transporter	MDP0000320480	
	Monosaccharide transporter	MDP0000485591	
	Hexose transporter		MDP0000216376, MDP0000219430
	Polyol transporter	MDP0000239167, MDP0000251579, MDP0000841918	
	Sugar transporter	MDP0000219048, MDP0000318992	MDP0000266249

Genes were annotated according to their similarity with *Arabidospis* genes (TAIR) and Mapman classification. Their deduced protein sequences where also search in ProtAnnDB, CAZy and Peroxibase databases

### Integrative analysis

Gene expression networks were realized within each cluster (Additional file 4). Gene correlation in cluster A yielded one large network composed of 70 genes (*r* > 0.7). A subset of genes in the network showed strong correlations (*P* < 0.05, *r* > 0.9) and was centred on a gene annotated for a glycoside hydrolase belonging to family 9 (GH9) grouping mainly glucanases (MDP0000131397). This subset was composed of GH9, β-glucosidase (MDP0000140817), β-galactosidase (MDP0000899966), XTH (MDP0000378203), FLA (MDP0000525641), AGP (MDP0000893240), peroxidase (MDP0000221335) and sugar transporter (MDP0000318992). Another subset containing CSLA (MDP0000717000), FLA (MDP0000658332) and β-galactosidase (MDP0000310582) also significantly correlated (*P* < 0.05, *r* > 0.9). Two small correlation networks were drawn for genes in cluster B (Additional file 4). Two gene networks showed significant correlations (*P* < 0.05, *r* > 0.7). One showed correlations between genes encoding pectin-degrading enzymes such as PG and pectin esterases (MDP0000249285; MDP0000251256; MDP0000252508; MDP0000287234) and genes encoding transporters (MDP0000216376; MDP000219430; MDP0000266249; MDP0000403872). The other network showed strong correlations (*P* < 0.05, *r* > 0.8) between genes encoding expansin like A (MDP0000906812), expansin like B (MDP0000214811; MDP0000292477), β-galactosidase (MDP0000416548) and peroxidase (MDP0000142485).

Transcriptomic profiles were tentatively correlated with the cell wall monosaccharides contents and the oligosaccharides enzymatically released from hemicelluloses in order to reveal concomitant events (Additional file 5). Total monosaccharide contents in AIM (as %NSP) were considered, except for arabinose and rhamnose whose content did not change (Table 1). Oligosaccharides relative contents in glucanase digests were also considered as markers of hemicellulose structural changes (Fig. 1b).

Glucose and uronic acids contents were the least correlated with the selected gene expression levels. Expression profiles of cluster A genes expressed during early developmental stages were positively correlated with galactose and mannose contents, as well as oligosaccharides content attributed to mannans (Hex4a1, Hex4a2, Hex5a1, Hex5a2, Hex6a1, Hex7a1 and Hex8a1) and xylans (Pen5U1m1a1, Pen4U1m1 and Pen4U1m1a1). They were also negatively correlated with fucose and xylose content as well as with oligosaccharides attributed to xyloglucans (XXG, XLG, GFG, XFG, XLXG, XXFG, XXFGa2, XLFGa2). The opposite correlations were observed with expression profiles from genes belonging to cluster B, showing higher expression at mature stages.

**Fig. 3:**
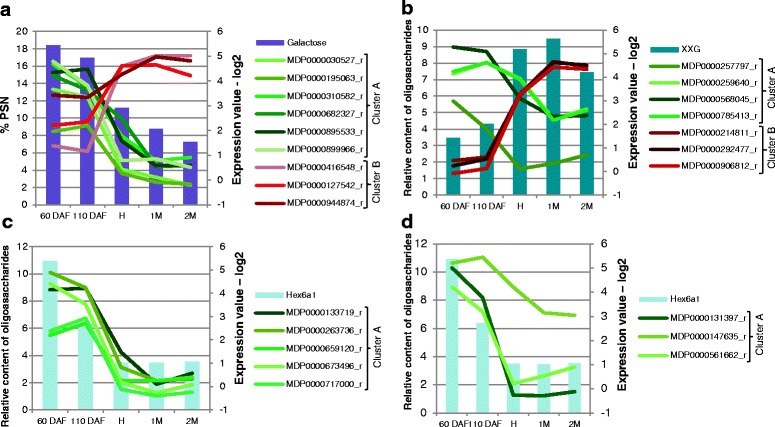
Expression level of genes correlated with monosaccharide content or oligosaccharides relative content MS from glucanase digest during apple development and ripening: **a** β-galactosidases and galactose content, **b** expansins and XXG, **c** cellulose synthases like A and Hex6a1, **d** glycoside hydrolases family 9 (glucanase) and Hex6a1. Green bold lines correspond to genes expressed during early apple development and red bold lines correspond to genes expressed during apple ripening. 60 DAF: 60 days after flowering, 110 DAF: 110 days after flowering, H: harvest, 1 M: 1 month of cold storage, 2 M: 2 months of cold storage

Strong correlations were observed between expression profiles of β-galactosidases and galactose content (Fig. 3a), between expression profiles of expansins and the structure XXG of xyloglucans (Fig. 3b), between expression profiles of cellulose synthase like-A and glycoside hydrolase family 9 and the structure Hex6a1 of mannans during apple development and ripening (Fig. 3c and d, respectively).

## Discussion

Modifications in the chemical composition of apple cell wall polysaccharides during fruit development and ripening have already been described [6, 9, 10, 22, 30] as well as enzymes and genes expression involved in ripening [14, 16, 17, 23, 26, 27, 39, 40]. However a more detailed view of genes potentially involved in cell wall polysaccharide chemical composition, structure, water flux during apple development and storage provides insights into the mechanisms affecting texture characteristics and highlights novel candidate genes involved in these processes.

### A dual approach to characterize apple cell wall dynamic

Biochemical cell wall analyses were done to assess the changes in polysaccharide composition and particularly that of hemicelluloses fine structure during apple fruit development. For the latter analyses, pectin was partially removed by water washes and pectinolytic enzymes as it was reported to mask hemicelluloses [41]. Our results showed that compared with previous studies of XGos profiles (Fig. 1), pectin in apple parenchyma cell wall did not have a major impact on hemicellulose accessibility to enzymes [7, 33, 42]. Furthermore, the changes observed by MALDI-TOF MS in the relative proportion of GgM oligomers in the glucanase hydrolysate, followed the decrease in mannose content in the cell wall of the fruit in development. Although these observations pointed to some degree of representativeness of the cell wall hemicelluloses enzymatic profiling, the hydrolyzates composition likely reflected readily accessible structures and not those in strong interaction. Additionally, the endogenous modifications of polysaccharides structure and access during fruit development possibly affected the enzymatic hydrolyzates composition.

Transcriptomic analysis provided access to genes encoding specific proteins and enzymes related to cell wall construction and remodelling during apple development, ripening and cold storage. Genes were selected according to their annotations from different databases but their respective biochemical activities remain to be characterized. Genome-wide expression analysis of apple fruit development has already revealed the coordination between gene expressions with specific fruit developmental stages from floral bud to ripe fruit [1, 43, 44]. Genes expressed during early fruit development are mainly involved in cell proliferation and expansion [43, 44]. Recently, this approach revealed that the down regulation of *MdPME2*, an early-expressed pectin methylesterase-coding gene during fruit development was linked to the apparition of mealiness, during fruit cold storage [21]. Several other functional categories have also been reported, such as solute transport and cell wall metabolism [45].

The present analysis confirmed that the number of transcripts detected was similar from early apple development to harvest stage and that it was not affected up to 2 months after cold storage [36]. However, remarkably more cell wall-related genes were specific to early developmental stages than to ripening and storage phases. This could be explained by the fact that analyses were carried out on distinct genotypes with different fruit texture evolution after harvest (Additional file 6). This difference in gene expression profiles highlights the plasticity of the genome with different expression time-frames and/or other genetic/environmental factors affecting markedly metabolic pathways during the ripening process. Indeed, variations in transcript profiles already observed between different apple genotypes support a genetic dependent regulation of fruit growth and ripening [43, 46].

### Pectin modification during fruit development and cold storage

During early apple development, genes involved in pectin metabolism were co-expressed with genes involved in hemicellulose metabolism and their remodelling by XTH (Table 4, Additional files 3 and 4). Concomitant expression of genes involved in biosynthetic and degradation functions is in line with observations that hydrolytic mechanisms are required to achieve a proper cell wall polysaccharide synthesis and organ development [47–49].

Changes in cell wall composition were observed as expected during fruit development and ripening, such as a decrease in galactose content [10, 11, 30, 50]. The increase in xylose, fucose and uronic acids likely resulted from both the cell wall enrichment in XyG and pectin depletion in neutral side-chains. The presence of uronic acids in the water washes of apple AIM at the ripening stage supports the hypothesis that HG depolymerization by PG contributes to the decrease of fruit firmness [14, 20, 51]. The removal of methyl esters facilitates PG action [3, 52] but also favours cell adhesion and the rigidity of pectin network [53]. In peach and tomato, pectin methyl-esterification decreased during development and ripening concomitantly with the increased activity of PME [52, 54, 55]. In apple, PME activity was reported to decrease during ripening [14, 17]. In this study, 2 pectin esterases coding gene (MDP0000177299, MDP0000212502) showed a decreased expression during apple development and ripening. However, as previously reported [10, 19, 21], no global significant variation was observed in methyl ester content of pectin during apple ripening. It was suggested that these enzymes could have a very local activity, at tricellular junctions, whose effect could not be evaluated at the whole fruit level [21]. Acetyl-esterification is also a common feature of apple pectin [56] and may be the target for the ill-defined function of these esterases. The latter may contribute to the significant decrease in the global cell wall acetyl esterification observed between 110 DAF and 2 M.

The specific expression of a GAUT coding gene (MDP0000609623) was also noticed during the ripening phases, as well as a significant increase of UA content. This suggests that new pectin could be incorporated into cell wall even after the developmental stages. This is in accordance with the observation that early ripening tomato has still cell wall synthetic capacities while being disassembled [57, 58].

### Hemicellulose changes during fruit development and cold storage ripening

The semi-quantitative variations of hemicellulose structural domains observed during fruit development (Fig. 1) were in accordance with observations made on other plant organs or fruits [59, 60]. This variation suggests different roles for different XyG fine structures in relation with cell wall expansion and extensibility mechanical properties by yet unclear mechanisms [61]. In several fruits, including apple, distinct xyloglucan transglycosylase/hydrolases (XTH)-coding genes are expressed in young and mature fruits and are likely to contribute to XyG structure [23, 62, 63]. Particularly found expressed in the early development phases in this study, XTH gene expression was highly and positively correlated (*P*-value < 0.01) with the expression of glycoside hydrolase GH9 (MDP0000131397), β-glucosidase (MDP0000140817), β-galactosidase (MDP0000899966), glycoproteins (FLA: MDP0000525641, AGP: MDP0000893240), peroxidase (MDP0000221335) and sugar transporter (MDP0000318992) (Additional file 4). It points out the key role of XyG in the cell wall dynamic together with other cell wall events including remodelling of cell wall polysaccharides by hydrolases, oxidative reactions and likely cell turgor regulation. Indeed, xyloglucans have been shown to be involved in cell wall mechanics, acting on the stability of microtubule cytoskeleton and the cellulose microfibrils biosynthesis and organization [64]. The concomitant expression of FLA genes (MDP0000525641, MDP0000658332) with XyG remodelling suggests that the proposed cell adhesion and plant mechanical implications of FLA proteins [65, 66] associate specific xyloglucan structures. Genes potentially coding a dual α-L-arabinofuranosidase/α -xylosidase (MDP0000208161) and α-L-fucosidase (MDP0000166406) were also highly expressed during early apple development. These genes should be further characterized as they may be involved in developmental regulation of XyG structure [67]. In addition, the xylosidase/arabinofuranosidase may also be implied in the remodelling of arabinan side chains of pectin, glucuronoarabinoxylan and/or AGP structures.

The present study revealed that galactoglucomannan (GgM), a minor component of apple hemicellulose, undergoes fine structural changes during early fruit development. It co-occurred with the high expression of one CSLA gene (MDP0000673496) (Additional file 5), whose *Arabidopsis* homolog was shown to encode mannan synthase [68] and has a potential key role on cell expansion [69]. In particular, this CSLA gene expression was positively correlated with the detection of the Hex6a1 structure in glucanase digest (Fig. 3c) and with the expression of the glycoside hydrolase GH9 gene (MDP0000131397) (Fig. 3d). The strong correlation of one CSLA (MDP0000717000) gene expression with the β-galactosidase expression profile (MDP0000310582) (*P*-value < 0.01) (Additional file 4) suggests that β-galactosidases may also be involved in the control of GgM synthesis and deposition in the wall. The function of GgM in primary walls remains unclear. Their potential interactions with cellulose [70] make them a candidate to control microfibrils aggregation and thus cell wall expansion [61]. This is consistent with the existence, at least in tomato, of mannan-degrading enzymes with transglycosylase activity (MTH) similar to XTH activity [71].

The large amount of mannose and glucose released by the combined action of pectin lyase and rhamnogalacturonase (PS; Table 3B) suggests that GgM could be associated with pectin. Such an association would also be affected by ripening as the amount of glucose decreased in 2 M samples when compared with 60DAF. The specific timing of GgM biosynthesis in early fruit development and metabolism as fruit enlarges points to a specific role most likely during and/or just after cell division with an implication in cell-cell adhesion as in tomato [72] and/or in the first rapid firmness decline observed during early fruit development [73].

Xylanase profiling did not show fine structural modifications of the minor glucuronoarabinoxylan (GAX) content in apple. This was likely due to the partial hydrolysis of the GAX by the xylanase contaminating the mannanase used in the previous hydrolysis step but also probably due to hindrance of the binding/active sites by xylan substitutions. Indeed, the xylanase activity contained in the following glucanase was able to release some more xylan oligomers. Overall, the mannanase- and glucanase-released xylo-oligomers revealed that GAX was particularly present at 60DAF and 110DAF, with different fine structures. There is no information on fine structure modifications of xylan-based polysaccharides in other fruits. As for GgM, the function of GAX in apple remains to be established. In *Arabidopsis*, xylan can be linked to pectin through AGP [74] and in tomato glucuronoxylan (GUX) are partly linked to GgM [75]. The identification of arabinose, galactose and rhamnose in the products of mannanase and xylanase hydrolysis supports the proposed connections between GAX and the AGP/pectin RGI complex. As with XTH and MTH, xylanase with hydrolase but also transglycosylase activity exists in fruit [76], opening the way for xylan remodelling mechanisms. Since tomato GUX were located in cell wall lining the intercellular spaces [72], it would be of interest to search for apple GAX in a similar location and test their involvement in the formation of large intercellular spaces in fruit flesh during cell expansion.

Expansins are also important proteins for loosening XyG-cellulose interactions during cell expansion [77, 78]. They have been found in developing fruit, such as tomato [79], pear [80], strawberry [81] and apple [82]. Our results showed that 4 expansin (EXPA) or expansin-like (EXLA) genes (MDP0000257797, MDP0000259640, MDP0000785413, and MDP0000568045) were specifically more expressed during early fruit development (Table 4; Additional file 3). Their deduced protein sequence had various level of similarity with expansin domains from others fleshy fruit [79–81, 83] (Additional file 7). In contrast, the protein sequence from the 3 expansin-like genes (EXL) (MDP0000214811, MDP0000292477, MDP0000906812) identified during fruit ripening was very different, suggesting different biochemical characteristics and biological functions (Additional file 7). Their gene expression profiles were correlated with oligosaccharides XXG relative content (Fig. 3b; Additional file 5), suggesting a target preference for this fine structure, or their involvement in cell wall integration of new XXG structures. Expansin genes (MDP0000214811; MDP0000292477; MDP0000906812) expression was also correlated with that of one β-galactosidase (MDP0000416548). This observation suggests that galactosylation of XyG structure may be involved in the recognition of XyG/cellulose complex by expansin.

### Two groups of β-galactosidases

β-galactosidases are common highly active enzymes during apple development and particularly during the ripening stage [6, 19, 25, 39, 40]. In the present study, 11 genes coding β-galactosidases were expressed during early apple development and had glycoside hydrolase 35 domain (GH35). They are similar to those identified in tomato and Japanese pear, but different from the *Arabidopsis* AtBGAL10 acting on xyloglucans [84] (Additional file 7). In particular, 7 of these apple β-galactosidases were very similar to PpGAL5, PpGAL6, and PpGAL7 whose expression was at their highest in expanding fruit but decreased drastically upon the onset of ripening [85]. These enzymes can potentially target several cell wall structures, such as AGP, pectic RGI galactan side-chains, xyloglucan or galactoglucomannans. The positive correlation of the 11 apple β-galactosidases expression pattern with galactose content (Fig. 3a, Additional file 5) suggests that RGI galactan side chains hydrolysis might not be their target unless these enzymes act also as transglycosylases as suggested by Franková and Fry [86]. In such a case, these enzymes would have more complex functions in the remodelling of cell wall polysaccharides. They might control pectin and hemicellulose polysaccharides interactions with cellulose through the modulation of their side chains structure [87–89], or regulate remodelling enzymes such as XTH/XET by modifying XyG galactosylation [90–92]. In any case, these glycosidases appear central in the remodelling of cell wall along with cell expansion.

Three other β-galactosidase genes were observed preferentially expressed during apple ripening (Fig. 3a). These β-galactosidases gene expression profiles were weakly and negatively correlated with galactose content (Additional file 5). Two of them (MD0000416548 and MD0000127542) corresponded to the up regulated β-galactosidase genes (Mdβ-GAL1 and Mdβ-GAL2) during apple storage in controlled or regular atmosphere at 1 °C [93]. Protein analysis showed that they were highly similar to the ripening specific pear proteins *PpGAL4* and *PpGAL1*, respectively (Additional file 7), which suggests that they may play similar roles in apple and pear ripening [40, 94, 95] in pruning of RGI galactan side chains [12, 96]. Ripening-specific β-galactosidase gene expression has also been reported in tomato [97] and down-regulation of one of them, TBG4, resulted in reduced fruit softening [98].

These results emphasize the complexity of the β-galactosidase family which merits further studies to assess if it could be split into two groups, one principally acting on the regulation of cell wall polysaccharides and glycoproteins galactosylation, and another one mainly pruning pectin RGI side chains.

### Potential others actors in cell wall dynamics

Peroxidases participate in a range of physiological processes [99], such as cell wall degradation by cleaving polysaccharides bonds through the generation of reactive oxygen species (ROS) [100–104]. Genes encoding peroxidases were found mostly expressed during early apple development. Some of them had expression profiles correlated with those from proteins and enzymes involved in biosynthesis, remodelling and degradation of cell wall polysaccharides. These results support a role for these enzymes in cell development as reported for *Arabidospis* cell root elongation [105] but their mechanism of action remains unclear. Indeed, beside polysaccharide degradation, certain ROS can inversely contribute to cell wall cross-linking and therefore limiting cell expansion [106]. Results from this study suggest that the regulation of apoplastic ROS production is important for cell wall biosynthesis and modifications during apple development.

During fruit growth and ripening, changes of internal turgor pressure lead to modifications of tissue mechanical properties [4, 8, 107]. Transporters have a major role in the regulation of turgor pressure by acting on apoplastic/cell solutes concentration and pH that affect osmotic pressure [2, 108, 109]. As previously reported [43, 44], genes encoding ions and sugar transporters were found highly expressed during early developmental phases (Table 4; Additional file 3). Their expression was highly correlated with genes involved in polysaccharides biosynthesis and degradation. Four genes encoding ion and sugar channels/transporters were preferentially expressed during apple ripening (Table 4; Additional file 3) and correlated with genes related to pectin degradation (Additional file 4). These early and late expressed transporter genes require further studies with regard to their implication in the regulation of turgor pressure during fruit expansion and ripening. During ripening, apple softening is known to involve a decrease in cell turgor pressure [8]. It would be of interest to assess the role of the hexose/sugar transporter genes expressed during late phases on the fate of the phloem downloaded sugars in the apoplast [110] with regard to the change in water compartmentalization observed in ripening apples [29]. Regulation of osmolytes concentration in ripening apples may contribute to changes in cell turgor pressure and loss of fruit firmness as proposed for grape berries [111]. This mechanism concomitant with cell wall polysaccharide remodelling and degradation may also participate in the elaboration of different apple textures, such as those perceived as mealiness and meltiness.

## Conclusion

In addition to known cell wall pectin changes during apple fruit development and cold storage, this study revealed changes in hemicellulose fine structure particularly during early fruit development. The associated characterization of the transcriptome highlighted genes potentially involved in hemicellulose metabolism and the changes observed. At the ripening stage, the low number of genes identified was more specific of pectin metabolism. This study also pointed to β-galactosidases whose roles during fruit growth and ripening remain to be characterized in regard to the targeted substrates and molecular function. Correlations observed between expression profiles from ion and sugar transporters, peroxidases and cell wall-related genes open the way to further studies on the interplay between cell wall assembly/disassembly mechanisms and cell turgor regulation during fruit development.

## Materials and methods

### Plant material

Eight hybrids (H074, H097, I016, I062, I095, V034, V083 and W029) from the ‘HIVW’ segregate population resulting from the cross between X3259 and X3263 done in our laboratory at INRA Angers [112, 113] were grown on 2 experimental plots (PH and P12) connected to the INRA laboratory at Bois l’Abbé domain (INRA, Angers). Fruits were collected from the middle and outside parts of the tree canopy at 60 and 110 days after flowering (DAF), and optimum maturity (Harvest, H). Skin colour and starch index (7–8) were used to evaluate fruit maturity at harvest [114]. Fruit collected at optimum maturity were also stored in a cold room at 1 °C for 1 month (1 M) and 2 months (2 M) before sampling. For RNA extraction, outer cortex from 5 fruits was sampled and frozen in liquid nitrogen immediately after collection or after 24 h at room temperature for 1 M and 2 M samples. For biochemical analyses, the cortex tissue of one fruit from each genotype and each plot was sampled and frozen in liquid nitrogen immediately after collection or after 24 h at room temperature for 1 M and 2 M samples (three to five fruits frozen per genotype and per plot). Fruits were sampled in 2012 except 60 DAF and 110 DAF fruits from PH plot harvested in 2013. Fruit softening from harvest to 2 M was checked using an automated penetrometer (TA.XT.-PLUS, Stable Micro system) equipped with a 4 mm diameter convex probes as described by Galvez-Lopez et al. [7] (Additional file 6).

### Gene expression analyses

#### RNA extraction, amplification and microarray hybridization

Total RNAs were extracted using a CTAB extraction buffer from 3 g of frozen fruit flesh tissue ground in liquid nitrogen, as described in Nobile et al.*,* [15] and Rienth et al.*,* [115]. mRNAs were amplified, labelled and co-hybridized according to Celton et al.*,* [36] as following: aRNAs were produced with Message AmpII aRNA amplification kit (Ambion) from 200 ng of total RNA. Then, 5 μg of each aRNAs were retrotranscribed and labelled with either Cyanine-3 or Cyanine-5 fluorescent dye (Interchim, Montluçon, France). Labelled samples were combined as 30 pmol for each dye and co-hybridized to the Nimblegen microarray AryANE v1.0 containing 135,000 60-mers oligonucleotide probes as described in Celton et al.*,* [36]. Deva software (Nimblegen) was used to extract pair-data files from the scanned images, obtained using the MS200 microarray scanner (Roche Nimblegen).

Genotypes were associated in 4 pairs (I062/V083, V034/W029, H097/I095, I016/H074) for competitive hybridizations at each time point (60 DAF, 110 DAF, H, 1 M and 2 M). Two independent biological repeats were performed with fruits from PH or P12 plots and technical replicates with dye swap were included for a total of 38 arrays.

### Statistical analysis of microarray data

All statistical analyses were performed as described in Celton et al.*,* [116] using R software [117]. Briefly, for each intensity comparison data were normalized with the Lowess method between microarrays. Normalized intensity values were then subtracted from the background to provide an estimation of the transcript expression levels. A second normalization by the quantiles method was then performed on expression values from all comparisons using the normalize.quantiles function from the R package preprocessCore [118] (Bioconductor project). Differential expression analyses between the different time points were carried out using the lmFit function and the Bayes moderated t test using the R package LIMMA [119] from the Bioconductor project. Genes were considered differentially expressed if the *t*-test *P-*values of the samples were below 1 % between 60 DAF and 2 M. To determine number probes expressed, only signals from the most specific probes belonging to classes 1–3 were considered for subsequent analysis (96,120 probes) [36].

The microarray data have been submitted to the Gene Expression Omnibus database (http://www.ncbi.nlm.nih.gov/geo/) under the accession number GSE64079.

### RT-qPCR analyses

cDNA synthesis and qPCR were performed on total-RNA samples used for the microarray experiment as described in Segonne-Mikol et al.*,* [21]. Primers were designed for short and specific amplification of the microarray probe region from the selected sequences with Primer 3 plus software (http://www.bioinformatics.nl/cgi-bin/primer3plus/primer3plus.cgi/) (Additional file 2). Amplicons were sequenced once for each genotype to verify primer specificity. For each run, single product amplification was confirmed by melting curve analysis. The amplification efficiency was tested for each primer pairs using a dilution curve method over a 6 points dilution series (from 1.10^−1^ to 1.10^−6^) on a pool of cDNAs containing all genotypes and developmental stages included in the study. Primers pairs selected for further analysis have efficiency above 90 %. RT-qPCR was carried out for 7 genes at 2 different time points.

Based on the microarray results, three reference genes with similar expression level in all samples were selected to calculate a normalization factor: MDP0000645828 (GAPDH), MDP0000146514 and MDP0000207727 (respectively annotated as Protein prenylyltransferase superfamily protein and DC1 domain-containing protein). Relative expression level was calculated using the formula ∆Ct = (Ct_tag_-Ct_ref_) derived from the 2^−ΔΔ*C*^_T_ method, where Ct is the threshold cycle, tag is the target gene, and ref is the reference gene [120] (Additional file 3).

### Sequences analyses

Apple genes annotation based on sequence similarity with *Arabidopsis thaliana* were retrieve from the Genome Database for Rosaceae (GDR, https://www.rosaceae.org/). Mapman gene ontology was used for functional classification and enrichment Wilcoxon test (*P*-value < 0.05) [121].

Hierarchical clustering of differentially expressed genes was performed with Genesis software (http://www.aics.riken.jp/labs/cbrt/) [122] using the average linkage hierarchical clustering method as agglomeration rule, and the distance was the similarity between gene expression. Correlation between expression profiles from selected genes were carried out using the cor.test function and the Pearson’s test using the R Stats package [117]. The expression correlation between two genes was selected if *r* >0.7 and *P*-value <0.01. The gene networks were generated using Cytoscape software [123].

Proteins annotations and predictions for sub-cellular localization were retrieve from the ProtAnnDB database (http://www.polebio.scsv.ups-tlse.fr/ProtAnnDB/) [124]. Potential peroxidases sequences were also analyzed through PeroxiBase database (http://peroxibase.toulouse.inra.fr/index.php) [125]. Proteins sequences multiple alignment were performed using Kalign tool (http://msa.sbc.su.se/cgi-bin/msa.cgi) with default parameters [126]. All protein sequences were retrieve from GenBank (http://www.ncbi.nlm.nih.gov/genbank/).

### Cell wall analyses

#### Enzymes

Pectin lyase (PL) [EC 4.2.2.10] was purified from Peclyve (Lyven, France) [127]. Rhamnogalacturonase (RG) [EC 3.2.1.171] was from *Aspergillus aculeatus* (Novozyme, Denmark). Endo-1,4-β-mannanase [EC 3.2.1.78] was from *Aspergillus niger* (E-BMANN Megazyme, Ireland). Endo-1,4-β-xylanase [EC 3.2.1.8] was from *Trichoderma viride* (E-XYTR1 Megazyme) and endo-β-glucanase [EC 3.2.1.6] was from *Trichoderma* sp. (E-CELTR Megazyme) and contained low activity towards xylan and glucomannan.

### Preparation of cell wall material

A pool of the 8 genotypes at each date was realized by picking randomly one fruit per genotype, per date for each orchard. Each sample was cut and lyophilized. Each dried sample was reduced to a fine powder with a mortar and a pestle. Cell walls were prepared as alcohol insoluble material (AIM) using an automated solvent extractor (ASE™ 350, Thermo Scientific™ Dionex™) using 80 % ethanol at 100 °C during 15 min until the ethanol extract was free of soluble sugars. The AIM was dried at 40 °C under vacuum over P_2_O_5_. The AIM obtained was reduced in powder using a benchtop homogenizer (FastPrep, MP Biomedicals, USA) at a speed of 6.5 m.s^−1^ for 20 s.

### AIM sugar composition

Cell wall neutral sugars content were identified and quantified from 5 mg AIM by sulfuric acid hydrolysis according to Hoebler et al.*,* [128]. AIM was dispersed in 13 M sulfuric acid during 30 min at 30 °C under agitation and then hydrolyzed in 1 M sulfuric acid (2 h at 100 °C). Sugars were reduced to alditols with NaBH_4_ (100 mg mL^−1^, NH_3_ 3.7 N) during 1 h at 40 °C under agitation. Then alditols were acetylated using acetic anhydride and imidazole during 20 min at room temperature [129]. Alditol acetates recovered in dichloromethane were analysed by GC (TRACE GC ULTRA, Thermo Scientific, USA) using TG-225MS column (Thermo Scientific, USA). Standard solution of sugars and inositol as an internal standard was used for calibration. Uronic acids content was quantified using the metahydroxydiphenyl colorimetric method [94, 97] with galacturonic acid as a standard.

### AIM starch content

To quantify starch content in cell wall material, AIM (10 mg) was incubated overnight at room temperature in 200 μL of MOPS (50 mM, pH7) followed by 5 min at 120 °C. Commercial thermostable α-amylase from *Bacillus licheniformis* (Megazyme, 3000 U mL^−1^) was added and the sample was incubated further for 6 min at 100 °C. After cooling, the sample was adjusted to pH 4.5 by addition of 400 μL of acetate buffer (200 mM, pH 4.5). It was further incubated for 30 min at 50 °C with commercial amyloglucosidase from *Aspergillus niger* (Megazyme, 3300 U mL^−1^). Glucose released was quantified in HPAEC-PAD using a CarboPac® PA1 column (4 mm × 250 mm, Thermo Scientific, USA), thermostated at 25 °C. An isocratic elution of 500 mM of NaOH was used at a flow rate 1 mL min^−1^. Rhamnose was used as an internal standard for calibration.

### Methyl and acetyl esters quantification in AIM

Methyl and acetyl esters was quantified from 5 mg AIM as described in Levigne et al.*,* [95]. Briefly, samples were saponified for 1 h at 4 °C in NaOH 1 N and CuSO_4_ 1 mg mL^−1^. After centrifugation (7400 g, 4 °C for 10 min), the supernatant was filtered (Alltech Maxi-Clean IC-H, Grace, USA) and analysed by HPLC with a C18 column (4 mm × 250 mm, 5 μm, Interchim, France), thermostated at 15 °C. An isocratic elution with 4 mM H_2_SO_4_ was used at a flow rate 1.0 mL min^−1^. Elution was monitored by differential refractometer (2414 refractive index detector, Waters, USA). Standard solution containing methanol, acetic acid and isopropanol as an internal standard was used for calibration. The degree of methylesterification (DM) of pectin was calculated as the number of moles of methanol per 100 moles of galacturonic acid (GalA).

### AIM polysaccharide structure profiling by enzymatic degradations

#### Enzymatic degradations

Pectin was partially removed from 5 mg AIM by deionized water (1 mL) at 40 °C under gentle agitation for 15 min followed by boiling for 10 min and centrifugation (15,300 *g*, 20 °C for 10 min). The supernatant was removed and the AIM pellet was suspended in deionized water (1 mL) and digested by a combination of pectin lyase and rhamnogalacturonase (0.12 U each) at 40 °C under gentle agitation for 3 h. After centrifugation (as above), the pellet was washed 3 times with 1 mL deionized water under agitation. Then sequential enzymatic digestion was applied on the pellet with endo-1,4-β-mannanase (10 U), endo-1,4-β-xylanase (25 U) and endo-β-glucanase (10 U) at 40 °C under gentle agitation for 17 h [7] (Additional file 8). The three water washes between enzymatic degradations were discarded. All supernatants were boiled 10 min prior to filtration (0.45 μm filter; Millex-Hv, PVDF, Millipore, France) and analysis by MALDI-TOF MS. Three replicates were realized for biological sample.

### Analysis of enzymatic degradation products

The degradation products were analysed by mass spectrometry MALDI. Each supernatant was combined with the ionic liquid matrix N,N-dimethylaniline/2,5-dihydroxybenzoic (DMA/DHB) [130] and dried on the MALDI polished steel plate. Three replicates were spotted per enzyme hydrolysate. MALDI-TOF MS analysis was performed in the positive mode on an Autoflex III (Bruker Daltonics, Germany) combined with a Smartbeam laser (355 nm, 1000 Hz). The instrument was externally calibrated using galactomannan oligomers (DP 3-9) of known mass. Spectra were recorded in the mass range m/z 500-3000. Ion masses and intensities were normalized on m/z 1085 (XXXG oligosaccharide), 731 (Hex4a1 oligosaccharide) and 655 (Pen3U1a1 oligosaccharide) for glucanase, mannanase and xylanase hydrolyzates, respectively. The nomenclature of xyloglucan was from Fry et al.*,* [131] extended to acetyl esters groups noted as a, followed by the number of ester groups in the oligosaccharide. In the oligosaccharides, Hex refers to hexose, Pen refers to pentose, U refers to uronic acid (UA), and m to methyl esters. For each letter, the following figure corresponds to the number of residues or substituents in the oligosaccharide.

### Statistical analysis

Principal component analyses were performed using the PCA function using the R Factominer package [132]. Analysis of variances of chemical data was performed using the anova function on R Stats package [117] and data were considered significantly different if *p* < 0.0001.

The correlation analysis between biochemical and transcriptomic data sets was performed using cor function and the Pearson’s test (*P*-value <0.01) from the R Stats package [117]. Expression data were grouped in three random pools and averaged (Pool1: V034, I062 and V083, Pool2: W029, I095 and H097, Pool3: H074 and I016), to match the three replicates available for biochemical data (Additional file 5).

### Availability of supporting data

The datasets supporting the conclusions of this article are included within the article and its additional files. The microarray dataset supporting the conclusions of this article is available in the Gene Expression Omnibus repository, under the accession number GSE64079 (http://www.ncbi.nlm.nih.gov/geo/).

## Additional files

Additional file 1: Differentially expressed genes during apple development and ripening. Expression data for differentially expressed genes between 60DAF and 2 M, displaying similar expression patterns for the eight genotypes (H074, H097, I016, I062, I095, V034, V083, W029) in both plots (PH and P12). Genes relatively more expressed during early fruit development (60 and 110 DAF) or during fruit maturation and cold storage (H, 1 M and 2 M) were respectively grouped in cluster A and B. Five time points are 60 days after flowering (60 DAF), 110 days after flowering (110 DAF), harvest (H), 1 month and 2 months of cold storage (1 M and 2 M). (XLSX 4945 kb) Additional file 2: Validation of microarrays results by RT-qPCR. S2-A) List of primers used for RT-qPCR. S2-B) Validation of microarrays results by quantitative real-time PCR (qRT-PCR) between 60 days after flowering (60 DAF) and 2 months of cold storage (2 M) for 4 genotypes grown on plot PH or P12. The three last genes are the reference genes used to calculate a normalization factor. Ratios were calculated on the normalized data from microarray analysis (log2 ratio) and as normalized expression for qRT-PCR (Ct ratio). Correlations between qRT-PCR Ct ratios and microarray log2 ratios are shown, along with Pearson correlation coefficient. (XLSX 18 kb) Additional file 3: Cell wall related selected genes and proteins annotation. Selection of 115 genes potentially involved in the cell wall dynamic during apple development, maturation and cold storage. Gene annotation was deduced from sequence similarity with Arabidopsis genes (TAIR, MapMan). Protein annotation was search in ProtannDB, CAZy and Peroxibase data bases. CAZy annotations: glycosyl transferases (GT), glycosyl hydrolase (GH), polysaccharide lyase (PL), carbohydrate esterase (CE), auxiliary activity (AA). The sub-cellular localization of the deduced proteins was predicted using ProtAnnDB and Peroxibase databases. Number of protein transmembrane domains was search using tmhmm. (XLSX 154 kb) Additional file 4: Co-expression networks of clusters A and B. The distance between 2 genes corresponds to level of correlation, the more the genes expression profiles are correlated, the shorter is the distance. The colour code indicates the gene functional category according to the curated annotation (Additional file 3). (PPTX 731 kb) Additional file 5: Heatmap of data sets correlation. Heatmap of correlation between selected genes expression profiles and monosaccharide content of cell wall (%NSP) or oligosaccharides relative content in glucanase digest during apple development and ripening. Positive and negative correlations are respectively shown in red and blue colours. (XLSX 28 kb) Additional file 6: Fruit firmness evolution during cold storage. Fruit firmness was evaluated by penetrometry from harvest (H) to 2 months of cold storage (2 M). Assessment of firmness was performed on the opposite sides of each fruit in the blush and shaded regions. Force in Newtons (N) was measured at 7 mm of displacement. The bold and dash lines are respectively associated with plot PH and P12. (PPTX 81 kb) Additional file 7: Matrix of identity for β-galactosidase glycosyl hydrolase 35 domain and expansin DPBB and Pollen allergen domains. S7-A: matrix of identity for β-galactosidase glycosyl hydrolase 35 domains. S7-B: matrix of identity for expansin DPBB (double-psi beta-barrel) and Pollen allergen domains. (XLSX 26 kb) Additional file 8: Schema of treatments was applied on AIM. (PPTX 63 kb)

## Abbreviations

1 M: One month of cold storage
2 M: Two months of cold storage
AGP: Arabinogalactan protein
AIM: Alcohol insoluble material
Ara: Arabinose
Fuc: Fucose
AS: Antisense
CSLA: Cellulose synthase-like A
DAF: Days after flowering
DM: Degree of methylesterification
EXLA: Expansin-like A
EXLB: Expansin-like B
EXPA: α-expansin
EXPB: β-expansin
FLA: Fasciclin like arabinogalactan protein
Gal: Galactose
GalA: Acid galacturonic
GATL: Galacturonosyltransferase-like
GAUT: Galacturonosyltransferase
GAX: Glucuronoarabinoxylan
GgM: Galactoglucomannan
GH: Glycoside hydrolase
Glc: Glucose
GT: Glycosyltransferase
GUX: Glucuronoxylan
H: Harvest
HG: Homogalacturonan
HRGP: Hydroxyproline-rich glycoproteins
Man: Mannose
MTH: Mannan endotransglucosylase/hydrolase
NS: Neutral sugars
NSP: Non-starch polysaccharides
PG: Polygalacturonase
PME: Pectin methylesterase
PS: Pectin soluble
RGI: Rhamnogalacturonan I
RGII: Rhamnogalacturonan II
Rha: Rhamnose
ROS: Reactive oxygen species
UA: Uronic acids
WS: Water soluble
XTH: Xyloglucan endotransglucosylase/hydrolase
XyG: Xyloglucan
XyGos: Xyloglucan oligosaccharides
Xyl: Xylose

## References

[1] Janssen BJ, Thodey K, Schaffer RJ, Alba R, Balakrishnan L, Bishop R, Bowen JH, Crowhurst RN, Gleave AP, Ledger S, McArtney S, Pichler FB, Snowden KC, Ward S (2008). Global gene expression analysis of apple fruit development from the floral bud to ripe fruit. BMC Plant Biol.

[2] Almeida DPF, Huber DJ (1999). Apoplastic pH and inorganic ion levels in tomato fruit: a potential means for regulation of cell wall metabolism during ripening. Physiol Plant.

[3] Toivonen PMA, Brummell DA (2008). Biochemical bases of appearance and texture changes in fresh-cut fruit and vegetables. Postharvest Biol Technol.

[4] Shackel KA, Greve C, Labavitch JM, Ahmadi H (1991). Cell turgor changes associated with ripening in tomato pericarp tissue. Plant Physiol.

[5] Saladié M, Matas AJ, Isaacson T, Jenks MA, Goodwin SM, Niklas KJ, Xiaolin R, Labavitch JM, Shackel KA, Fernie AR, Lytovchenko A, O’Neill MA, Watkins CB, Rose JKC (2007). A reevaluation of the key factors that influence tomato fruit softening and integrity. Plant Physiol.

[6] Brummell DA (2006). Cell wall disassembly in ripening fruit. Funct Plant Biol.

[7] Galvez-Lopez D, Laurens F, Quéméner B, Lahaye M (2011). Variability of cell wall polysaccharides composition and hemicellulose enzymatic profile in an apple progeny. Int J Biol Macromol.

[8] Tong C, Krueger D, Vickers Z, Bedford D, Luby J, El-Shiekh A, Shackel KA, Ahmadi H (1999). Comparison of softening-related changes during storage of ‘Honeycrisp’ apple, its parents, and ‘Delicious’. J Amer Soc Hort Sci.

[9] Nelmes BJ, Preston RD (1968). Wall development in apple fruits: a study of the life history of a parenchyma cell. J Exp Bot.

[10] Fischer M, Amado R (1994). Changes in the pectic substances of apples during development and postharvest ripening. Part 1: analysis of the alcohol-insoluble residue. Carbohydr Polym.

[11] Gross KC, Sams CE (1984). Changes in cell wall neutral sugar composition during fruit ripening: a species survey. Phytochemistry.

[12] Peña MJ, Carpita NC (2004). Loss of highly branched arabinans and debranching of rhamnogalacturonan I accompany loss of firm texture and cell separation during prolonged storage of apple. Plant Physiol.

[13] Redgwell RJ, Fischer M, Kendal E, MacRae EA (1997). Galactose loss and fruit ripening: high-molecular-weight arabinogalactans in the pectic polysaccharides of fruit cell walls. Planta.

[14] Goulao LF, Oliveira CM (2008). Cell wall modifications during fruit ripening: when a fruit is not the fruit. Trends Food Sci Technol.

[15] Nobile PM, Wattebled F, Quecini V, Girardi CL, Lormeau M, Laurens F (2011). Identification of a novel α-L-arabinofuranosidase gene associated with mealiness in apple. J Exp Bot.

[16] Wei J, Ma F, Shi S, Qi X, Zhu X, Yuan J (2010). Changes and postharvest regulation of activity and gene expression of enzymes related to cell wall degradation in ripening apple fruit. Postharvest Biol Technol.

[17] Goulao LF, Santos J, de Sousa I, Oliveira CM (2007). Patterns of enzymatic activity of cell wall-modifying enzymes during growth and ripening of apples. Postharvest Biol Technol.

[18] Jarvis MC, Briggs SPH, Knox JP (2003). Intercellular adhesion and cell separation in plants. Plant Cell Environ.

[19] Ng JKT, Schröder R, Brummell DA, Sutherland PW, Hallett IC, Smith BG, Melton LD, Johnston JW (2015). Lower cell wall pectin solubilisation and galactose loss during early fruit development in apple (Malus x domestica) cultivar “Scifresh” are associated with slower softening rate. J Plant Physiol.

[20] Atkinson RG, Sutherland PW, Johnston SL, Gunaseelan K, Hallett IC, Mitra D, Brummell DA, Schröder R, Johnston JW, Schaffer RJ (2012). Down-regulation of POLYGALACTURONASE1 alters firmness, tensile strength and water loss in apple (Malus x domestica) fruit. BMC Plant Biol.

[21] Segonne-Mikol S, Bruneau M, Celton J-M, Le Gall S, Francin-Allami M, Juchaux M, Laurens F, Orsel M, Renou J-P (2014). Multiscale investigation of mealiness in apple: an atypical role for a pectin methylesterase during fruit maturation. BMC Plant Biol.

[22] Percy AE, Melton LD, Jameson PE (1997). Xyloglucan and hemicelluloses in the cell wall during apple fruit development and ripening. Plant Sci.

[23] Atkinson RG, Johnston SL, Yauk Y-K, Sharma NN, Schröder R (2009). Analysis of xyloglucan endotransglucosylase/hydrolase (XTH) gene families in kiwifruit and apple. Postharvest Biol Technol.

[24] Costa F, Van de Weg WE, Stella S, Dondini L, Pratesi D, Musacchi S, Sansavini S (2008). Map position and functional allelic diversity of Md-Exp7, a new putative expansin gene associated with fruit softening in apple (malus × domestica borkh.) and pear (pyrus communis). Tree Genet Genomes.

[25] Ireland HS, Gunaseelan K, Muddumage R, Tacken EJ, Putterill J, Johnston JW, Schaffer RJ (2014). Ethylene regulates apple (malus x domestica) fruit softening through a dose x time-dependent mechanism and through differential sensitivities and dependencies of cell wall-modifying genes. Plant Cell Physiol.

[26] Trujillo DI, Mann HS, Tong CBS (2011). Examination of expansin genes as related to apple fruit crispness. Tree Genet Genomes.

[27] Wakasa Y, Hatsuyama Y, Takahashi A, Sato T, Niizeki M, Harada T (2003). Divergent expression of six expansin genes during apple fruit ontogeny. Eur J Hortic Sci.

[28] Ting VJL, Silcock P, Bremer PJ, Biasioli F (2013). X-ray micro-computer tomographic method to visualize the microstructure of different apple cultivars. J Food Sci.

[29] Winisdorffer G, Musse M, Quellec S, Barbacci A, Le GS, Mariette F, Lahaye M (2015). Analysis of the dynamic mechanical properties of apple tissue and relationships with the intracellular water status, gas distribution, histological properties and chemical composition. Postharvest Biol Technol.

[30] Ng JKT, Schröder R, Sutherland PW, Hallett IC, Hall MI, Prakash R, Smith BG, Melton LD, Johnston JW (2013). Cell wall structures leading to cultivar differences in softening rates develop early during apple (malus × domestica) fruit growth. BMC Plant Biol.

[31] Khan AA, Vincent JFV (1993). Compressive stiffness and fracture properties of apple and potato parenchyma. J Texture Stud.

[32] Barreiro P, Moya A, Correa E, Ruiz-Altisent M, Fernández-Valle M, Peirs A, Wright KM, Hills BP (2002). Prospects for the rapid detection of mealiness in apples by nondestructive NMR relaxometry. Appl Magn Reson.

[33] Ray S, Vigouroux J, Quémener B, Bonnin E, Lahaye M (2014). Novel and diverse fine structures in LiCl-DMSO extracted apple hemicelluloses. Carbohydr Polym.

[34] Nara K, Ito S, Kato K, Kato Y (2004). Isolation of galactoglucomannan from apple hemicellulosic polysaccharides with binding capacity to cellulose. J Appl Glycosci.

[35] Voragen FGJ, Schols HA, Pilnik W (1986). Structural features of the hemicellulose polymers of apples. Z Lebensm Unters Forsch.

[36] Celton J-M, Gaillard S, Bruneau M, Pelletier S, Aubourg S, Martin-Magniette M-L, Navarro L, Laurens F, Renou J-P (2014). Widespread anti-sense transcription in apple is correlated with siRNA production and indicates a large potential for transcriptional and/or post-transcriptional control. New Phytol.

[37] San Clemente H, Jamet E (2015). WallProtDB, a database resource for plant cell wall proteomics. Plant Methods.

[38] Velasco R, Zharkikh A, Affourtit J, Dhingra A, Cestaro A, Kalyanaraman A, Fontana P, Bhatnagar SK, Troggio M, Pruss D, Salvi S, Pindo M, Baldi P, Castelletti S, Cavaiuolo M, Coppola G, Costa F, Cova V, Dal Ri A, Goremykin V, Komjanc M, Longhi S, Magnago P, Malacarne G, Malnoy M, Micheletti D, Moretto M, Perazzolli M, Si-Ammour A, Vezzulli S (2010). The genome of the domesticated apple (malus × domestica borkh.). Nat Genet.

[39] Gwanpua SG, Mellidou I, Boeckx J, Kyomugasho C, Bessemans N, Verlinden BE, Hertog MLATM, Hendrickx M, Nicolai B, Geeraerd AH. Expression analysis of candidate cell wall-related genes associated with changes in pectin biochemistry during postharvest apple softening. Postharvest Biol Technol. 2016.

[40] Harb J, Gapper NE, Giovannoni JJ, Watkins CB (2012). Molecular analysis of softening and ethylene synthesis and signaling pathways in a non-softening apple cultivar, “Honeycrisp” and a rapidly softening cultivar, “McIntosh.”. Postharvest Biol Technol.

[41] Marcus SE, Verhertbruggen Y, Hervé C, Ordaz-Ortiz JJ, Farkas V, Pedersen HL, Willats WGT, Knox JP (2008). Pectic homogalacturonan masks abundant sets of xyloglucan epitopes in plant cell walls. BMC Plant Biol.

[42] Renard CMGC, Lomax JA, Boon JJ (1992). Apple-fruit xyloglucans: a comparative study of enzyme digests of whole cell walls and of alkali-extracted xyloglucans. Carbohydr Res.

[43] Soria-Guerra RE, Rosales-Mendoza S, Gasic K, Wisniewski ME, Band M, Korban SS (2011). Gene expression is highly regulated in early developing fruit of apple. Plant Mol Biol Report.

[44] Lee Y-P, Yu G-H, Seo YS, Han SE, Choi Y-O, Kim D, Mok I-G, Kim WT, Sung S-K (2007). Microarray analysis of apple gene expression engaged in early fruit development. Plant Cell Rep.

[45] Soglio V, Costa F, Molthoff JW, Weemen-Hendriks WMJ, Schouten HJ, Gianfranceschi L (2009). Transcription analysis of apple fruit development using cDNA microarrays. Tree Genet Genomes.

[46] Zhu Y, Zheng P, Varanasi V, Shin S, Main D, Curry E, Mattheis JP (2012). Multiple plant hormones and cell wall metabolism regulate apple fruit maturation patterns and texture attributes. Tree Genet Genomes.

[47] Nicol F, His I, Jauneau A, Vernhettes S, Canut H, Höfte H (1998). A plasma membrane-bound putative endo-1,4-beta-D-glucanase is required for normal wall assembly and cell elongation in Arabidopsis. EMBO J.

[48] Wilson MH, Holman TJ, Sørensen I, Cancho-Sanchez E, Wells DM, Swarup R, Knox JP, Willats WGT, Ubeda-Tomás S, Holdsworth M, Bennett MJ, Vissenberg K, Hodgman TC (2015). Multi-omics analysis identifies genes mediating the extension of cell walls in the Arabidopsis thaliana root elongation zone. Front cell Dev Biol.

[49] Xiao C, Somerville C, Anderson CT (2014). POLYGALACTURONASE INVOLVED IN EXPANSION1 functions in cell elongation and flower development in Arabidopsis. Plant Cell.

[50] Massiot P, Baron A, Drilleau JF (1996). Effect of storage of apple on the enzymatic hydrolysis of cell wall polysaccharides. Carbohydr Polym.

[51] Costa F, Peace CP, Stella S, Serra S, Musacchi S, Bazzani M, Sansavini S, Van de Weg WE (2010). QTL dynamics for fruit firmness and softening around an ethylene-dependent polygalacturonase gene in apple (malus × domestica borkh.). J Exp Bot.

[52] Brummell DA, Dal Cin V, Crisosto CH, Labavitch JM (2004). Cell wall metabolism during maturation, ripening and senescence of peach fruit. J Exp Bot.

[53] Levesque-Tremblay G, Pelloux J, Braybrook SA, Müller K. Tuning of pectin methylesterification: consequences for cell wall biomechanics and development. Planta. 2015. 10.1007/s00425-015-2358-5 26168980

[54] Harriman RW, Tieman DM, Handa AK (1991). Molecular cloning of tomato pectin methylesterase gene and its expression in Rutgers, ripening inhibitor, nonripening, and never ripe tomato fruits. PLANT Physiol.

[55] Hyodo H, Terao A, Furukawa J, Sakamoto N, Yurimoto H, Satoh S, Iwai H (2013). Tissue specific localization of pectin-Ca^2+^ cross-linkages and pectin methyl-esterification during fruit ripening in tomato (Solanum lycopersicum). PLoS One.

[56] Schols HA, Voragen AGJ. Complex pectins: Structure elicudation using enzymes In: Visser J, Voragen AGJ, editors. Pectins and Pectinases. vol. 14. Amsterdam: Elsevier; 1996. p. 3–19.

[57] Greve LC, Labavitch JM (1991). Cell wall metabolism in ripening fruit : V. Analysis of cell wall synthesis in ripening tomato pericarp tissue using a D-[U-13C] glucose tracer and gas chromatography–mass spectrometry. Plant Physiol.

[58] Huysamer M, Greve LC, Labavitch JM (1997). Cell wall metabolism in ripening fruit. VIII. Cell wall composition and synthetic capacity of two regions of the outer pericarp of mature green and red ripe cv. Jackpot tomatoes. Physiol Plant.

[59] Pauly M, Qin Q, Greene H, Albersheim P, Darvill A, York WS (2001). Changes in the structure of xyloglucan during cell elongation. Planta.

[60] Lahaye M, Quemener B, Causse M, Seymour GB (2012). Hemicellulose fine structure is affected differently during ripening of tomato lines with contrasted texture. Int J Biol Macromol.

[61] Park YB, Cosgrove DJ (2015). Xyloglucan and its interactions with other components of the growing cell wall. Plant Cell Physiol.

[62] Han Y, Zhu Q, Zhang Z, Meng K, Hou Y, Ban Q, Suo J, Rao J (2015). Analysis of xyloglucan endotransglycosylase/hydrolase (XTH) genes and diverse roles of isoenzymes during persimmon fruit development and postharvest softening. PLoS One.

[63] Miedes E, Lorences EP (2009). Xyloglucan endotransglucosylase/hydrolases (XTHs) during tomato fruit growth and ripening. J Plant Physiol.

[64] Xiao C, Zhang T, Zheng Y, Cosgrove DJ, Anderson CT. Xyloglucan deficiency disrupts microtubule stability and cellulose biosynthesis in Arabidopsis, altering cell growth and morphogenesis. Plant Physiol. 2016;170:234–49. 10.1104/pp.15.01395 PMC4704587 26527657

[65] Johnson KL, Jones BJ, Bacic A, Schultz CJ (2003). The fasciclin-like arabinogalactan proteins of Arabidopsis. A multigene family of putative cell adhesion molecules. Plant Physiol.

[66] MacMillan CP, Mansfield SD, Stachurski ZH, Evans R, Southerton SG (2010). Fasciclin-like arabinogalactan proteins: specialization for stem biomechanics and cell wall architecture in Arabidopsis and Eucalyptus. Plant J.

[67] Günl M, Pauly M (2011). AXY3 encodes a α-xylosidase that impacts the structure and accessibility of the hemicellulose xyloglucan in Arabidopsis plant cell walls. Planta.

[68] Liepman AH, Wilkerson CG, Keegstra K (2005). Expression of cellulose synthase-like (Csl) genes in insect cells reveals that CslA family members encode mannan synthases. Proc Natl Acad Sci U S A.

[69] Goubet F, Misrahi A, Park SK, Zhang Z, Twell D, Dupree P (2003). AtCSLA7, a cellulose synthase-like putative glycosyltransferase, is important for pollen tube growth and embryogenesis in Arabidopsis. Plant Physiol.

[70] Melton LD, Smith BG, Ibrahim R, Schröder R (2009). Mannans in primary and secondary plant cell walls. New Zeal J For Sci.

[71] Schröder R, Atkinson RG, Redgwell RJ (2009). Re-interpreting the role of endo-beta-mannanases as mannan endotransglycosylase/hydrolases in the plant cell wall. Ann Bot.

[72] Ordaz-Ortiz JJ, Marcus SE, Paul Knox J (2009). Cell wall microstructure analysis implicates hemicellulose polysaccharides in cell adhesion in tomato fruit pericarp parenchyma. Mol Plant.

[73] Volz RK, Harker FR, Lang S (2003). Firmness decline in ‘Gala’ apple during fruit development. J Amer Soc Hort Sci.

[74] Tan L, Eberhard S, Pattathil S, Warder C, Glushka J, Yuan C, Hao Z, Zhu X, Avci U, Miller JS, Baldwin D, Pham C, Orlando R, Darvill A, Hahn MG, Kieliszewski MJ, Mohnen D (2013). An Arabidopsis cell wall proteoglycan consists of pectin and arabinoxylan covalently linked to an arabinogalactan protein. Plant Cell.

[75] Prakash R, Johnston SL, Boldingh HL, Redgwell RJ, Atkinson RG, Melton LD, Brummell DA, Schröder R (2012). Mannans in tomato fruit are not depolymerized during ripening despite the presence of endo-β-mannanase. J Plant Physiol.

[76] Johnston SL, Prakash R, Chen NJ, Kumagai MH, Turano HM, Cooney JM, Atkinson RG, Paull RE, Cheetamun R, Bacic A, Brummell DA, Schröder R (2013). An enzyme activity capable of endotransglycosylation of heteroxylan polysaccharides is present in plant primary cell walls. Planta.

[77] McQueen-Mason S (1992). Two endogenous proteins that induce cell wall extension in plants. Plant Cell.

[78] Cosgrove DJ (2015). Plant expansins: diversity and interactions with plant cell walls. Curr Opin Plant Biol.

[79] Brummell DA, Harpster MH, Dunsmuir P (1999). Differential expression of expansin gene family members during growth and ripening of tomato fruit. Plant Mol Biol.

[80] Hiwasa K, Rose JKC, Nakano R, Inaba A, Kubo Y (2003). Differential expression of seven alpha-expansin genes during growth and ripening of pear fruit. Physiol Plant.

[81] Harrison EP, McQueen-Mason SJ, Manning K (2001). Expression of six expansin genes in relation to extension activity in developing strawberry fruit. J Exp Bot.

[82] Zhang S, Xu R, Gao Z, Chen C, Jiang Z, Shu H (2014). A genome-wide analysis of the expansin genes in malus × domestica. Mol Genet Genomics.

[83] Hayama H, Ito A, Moriguchi T, Kashimura Y (2003). Identification of a new expansin gene closely associated with peach fruit softening. Postharvest Biol Technol.

[84] Sampedro J, Cosgrove DJ (2005). The expansin superfamily. Genome Biol.

[85] Tateishi A, Nagashima K, Mathooko FM, Mwaniki MW, Kubo Y, Inaba A, Yamaki S, Inoue H (2005). Differential expression of members of the β-galactosidase gene family during Japanese pear (Pyrus pyrifolia L.) fruit growth and on-tree ripening. J Am Soc Hortic Sci.

[86] Franková L, Fry SC (2013). Biochemistry and physiological roles of enzymes that “cut and paste” plant cell-wall polysaccharides. J Exp Bot.

[87] de Lima D, Buckeridge M (2001). Interaction between cellulose and storage xyloglucans: the influence of the degree of galactosylation. Carbohydr Polym.

[88] Zykwinska A, Thibault J-F, Ralet M-C (2007). Organization of pectic arabinan and galactan side chains in association with cellulose microfibrils in primary cell walls and related models envisaged. J Exp Bot.

[89] Lin D, Lopez-Sanchez P, Gidley MJ (2015). Binding of arabinan or galactan during cellulose synthesis is extensive and reversible. Carbohydr Polym.

[90] Maris A, Suslov D, Fry SC, Verbelen J-P, Vissenberg K (2009). Enzymic characterization of two recombinant xyloglucan endotransglucosylase/hydrolase (XTH) proteins of Arabidopsis and their effect on root growth and cell wall extension. J Exp Bot.

[91] Maris A, Kaewthai N, Eklöf JM, Miller JG, Brumer H, Fry SC, Verbelen J-P, Vissenberg K (2011). Differences in enzymic properties of five recombinant xyloglucan endotransglucosylase/hydrolase (XTH) proteins of Arabidopsis thaliana. J Exp Bot.

[92] Shi H, Kim Y, Guo Y, Stevenson B, Zhu J-K (2003). The Arabidopsis SOS5 locus encodes a putative cell surface adhesion protein and is required for normal cell expansion. Plant Cell.

[93] Gwanpua SG, Mellidou I, Boeckx J, Kyomugasho C, Bessemans N, Verlinden BE, Hertog MLATM, Hendrickx M, Nicolai BM, Geeraerd AH (2016). Expression analysis of candidate cell wall-related genes associated with changes in pectin biochemistry during postharvest apple softening. Postharvest Biol Technol.

[94] Thibault J-F (1979). Automatisation du dosage des substances pectiques par la méthode au meta-hydroxydiphenyl. Leb Technol.

[95] Levigne S, Thomas M, Ralet M-C, Quemener B, Thibault J-F (2002). Determination of the degrees of methylation and acetylation of pectins using a C18 column and internal standards. Food Hydrocoll.

[96] Fischer M, Arrigoni E, Amado R (1994). Changes in the pectic substances of apples during development and postharvest ripening. Part 2: analysis of the pectic fractions. Carbohydr Polym.

[97] Blumenkrantz N, Asboe-Hansen G (1973). New method for quantitative determination of uronic acids. Anal Biochem.

[98] Smith DL, Abbott JA, Gross KC (2002). Down-regulation of tomato beta-galactosidase 4 results in decreased fruit softening. Plant Physiol.

[99] Cosio C, Dunand C (2009). Specific functions of individual class III peroxidase genes. J Exp Bot.

[100] Brownleader MD, Ahmed N, Trevan M, Chaplin MF, Dey PM (1995). Purification and partial characterization of tomato extensin peroxidase. Plant Physiol.

[101] Liszkay A, Kenk B, Schopfer P (2003). Evidence for the involvement of cell wall peroxidase in the generation of hydroxyl radicals mediating extension growth. Planta.

[102] Dunand C, Tognolli M, Overney S, von Tobel L, de Meyer M, Simon P, Penel C (2002). Identification and characterisation of Ca2 + −pectate binding peroxidases in Arabidopsis thaliana. J Plant Physiol.

[103] Fry SC (1998). Oxidative scission of plant cell wall polysaccharides by ascorbate-induced hydroxyl radicals. Biochem J.

[104] Schopfer P (2001). Hydroxyl radical-induced cell-wall loosening in vitro and in vivo: implications for the control of elongation growth. Plant J.

[105] Passardi F, Tognolli M, De Meyer M, Penel C, Dunand C (2006). Two cell wall associated peroxidases from Arabidopsis influence root elongation. Planta.

[106] Passardi F, Penel C, Dunand C (2004). Performing the paradoxical: how plant peroxidases modify the cell wall. Trends Plant Sci.

[107] Castellarin SD, Gambetta GA, Wada H, Krasnow MN, Cramer GR, Peterlunger E, Shackel KA, Matthews MA (2015). Characterization of major ripening events during softening in grape: turgor, sugar accumulation, abscisic acid metabolism, colour development, and their relationship with growth. J Exp Bot.

[108] Grignon C, Sentenac H (1991). pH and ionic conditions in the apoplast. Annu Rev Plant Physiol Plant Mol Biol.

[109] Shiratake K, Martinoia E (2007). Transporters in fruit vacuoles. Plant Biotechnol.

[110] Zhang L-Y, Peng Y-B, Pelleschi-Travier S, Fan Y, Lu Y-F, Lu Y-M, Gao X-P, Shen Y-Y, Delrot S, Zhang D-P (2004). Evidence for apoplasmic phloem unloading in developing apple fruit. Plant Physiol.

[111] Wada H, Matthews MA, Shackel KA (2009). Seasonal pattern of apoplastic solute accumulation and loss of cell turgor during ripening of Vitis vinifera fruit under field conditions. J Exp Bot.

[112] Kouassi AB, Durel C-E, Costa F, Tartarini S, van de Weg E, Evans K, Fernandez-Fernandez F, Govan C, Boudichevskaja A, Dunemann F, Antofie A, Lateur M, Stankiewicz-Kosyl M, Soska A, Tomala K, Lewandowski M, Rutkovski K, Zurawicz E, Guerra W, Laurens F (2009). Estimation of genetic parameters and prediction of breeding values for apple fruit-quality traits using pedigreed plant material in Europe. Tree Genet Genomes.

[113] Ben Sadok I, Tiecher A, Galvez-Lopez D, Lahaye M, Lasserre-Zuber P, Bruneau M, Hanteville S, Robic R, Cournol R, Laurens F (2015). Apple fruit texture QTLs: year and cold storage effects on sensory and instrumental traits. Tree Genet Genomes.

[114] Pitts MJ, Cavalieri RP. Objective assessment of apple maturity based on starch location. Trans ASAE. 1988;31.

[115] Rienth M, Torregrosa L, Ardisson M, De Marchi R, Romieu C (2014). Versatile and efficient RNA extraction protocol for grapevine berry tissue, suited for next generation RNA sequencing. Aust J Grape Wine Res.

[116] Celton J-M, Dheilly E, Guillou M-C, Simonneau F, Juchaux M, Costes E, Laurens F, Renou J-P (2014). Additional amphivasal bundles in pedicel pith exacerbate central fruit dominance and induce self-thinning of lateral fruitlets in apple. Plant Physiol.

[117] R Development Core Team: R: A language and environment for statistical computing. 2013. https://www.r-project.org/.

[118] Bolstad BM (2001). Probe level quantile normalization of high density oligonucleotide array data.

[119] Smyth GK, Gentleman R, Carey V, Dudoit S, Irizarry R, Hubert W (2005). Limma: linear models for microarray data. Bioinformatics and computational biology solutions using R and bioconductor.

[120] Livak KJ, Schmittgen TD (2001). Analysis of relative gene expression data using real-time quantitative PCR and the 2(−delta delta C(T)) method. Methods.

[121] Usadel B, Nagel A, Thimm O, Redestig H, Blaesing OE, Palacios-Rojas N, Selbig J, Hannemann J, Piques MC, Steinhauser D, Scheible W-R, Gibon Y, Morcuende R, Weicht D, Meyer S, Stitt M (2005). Extension of the visualization tool MapMan to allow statistical analysis of arrays, display of corresponding genes, and comparison with known responses. Plant Physiol.

[122] Sturn A, Quackenbush J, Trajanoski Z (2002). Genesis. Bioinformatics.

[123] Shannon P, Markiel A, Ozier O, Baliga NS, Wang JT, Ramage D, Amin N, Schwikowski B, Ideker T (2003). Cytoscape: a software environment for integrated models of biomolecular interaction networks. Genome Res.

[124] Clemente HS, Pont-Lezica R, Jamet E (2009). Bioinformatics as a tool for assessing the quality of sub-cellular proteomic strategies and inferring functions of proteins: plant cell wall proteomics as a test case. Bioinform Biol Insights.

[125] Fawal N, Li Q, Savelli B, Brette M, Passaia G, Fabre M, Mathé C, Dunand C (2013). PeroxiBase: a database for large-scale evolutionary analysis of peroxidases. Nucleic Acids Res.

[126] Lassmann T, Sonnhammer ELL (2005). Kalign--an accurate and fast multiple sequence alignment algorithm. BMC Bioinformatics.

[127] Ralet M-C, Williams MAK, Tanhatan-Nasseri A, Ropartz D, Quemener B, Bonnin E (2012). Innovative enzymatic approach to resolve homogalacturonans based on their methylesterification pattern. Biomacromolecules.

[128] Hoebler C, Barry JL, David A, Delort-Laval J (1989). Rapid acid hydrolysis of plant cell wall polysaccharides and simplified quantitative determination of their neutral monosaccharides by gas–liquid chromatography. J Agric Food Chem.

[129] Englyst HN, Cummings JH (1988). Improved method for measurement of dietary fiber as non-starch polysaccharides in plant food. J Assoc Off Anal Chem.

[130] Ropartz D, Bodet P-E, Przybylski C, Gonnet F, Daniel R, Fer M, Helbert W, Bertrand D, Rogniaux H (2011). Performance evaluation on a wide set of matrix-assisted laser desorption ionization matrices for the detection of oligosaccharides in a high-throughput mass spectrometric screening of carbohydrate depolymerizing enzymes. Rapid Commun Mass Spectrom.

[131] Fry SC, York WS, Albersheim P, Darvill A, Hayashi T, Joseleau J-P, Kato Y, Lorences EP, Maclachlan GA, McNeil M, Mort AJ, Grant Reid JS, Seitz HU, Selvendran RR, Voragen AGJ, White AR (1993). An unambiguous nomenclature for xyloglucan-derived oligosaccharides. Physiol Plant.

[132] Lê S, Josse J, Husson F (2008). FactoMineR: an R package for multivariate analysis. J Stat Softw.

[133] Tuomivaara ST, Yaoi K, O'Neill MA, York WS (2015). Generation and structural validation of a library of diverse xyloglucan-derived oligosaccharides, including an update on xyloglucan nomenclature. Carbohydr Res.

