# Supplementary material for: Cell wall dynamics during apple development and storage involves hemicellulose modifications and related expressed genes
Source: BMC Plant Biol. 2016 Sep 15;16:201. doi: 10.1186/s12870-016-0887-0 (PMC5024441; doi:10.1186/s12870-016-0887-0)
Supplement: Additional file 4: — Co-expression networks of clusters A and B. The distance between 2 genes corresponds to level of correlation, the more the genes expression profiles are correlated, the shorter is the distance. The colour code indicates the gene functional category according to the curated annotation (Additional file 3). (PPTX 731 kb) [file 12870_2016_887_MOESM4_ESM.pptx]

## Slide 1
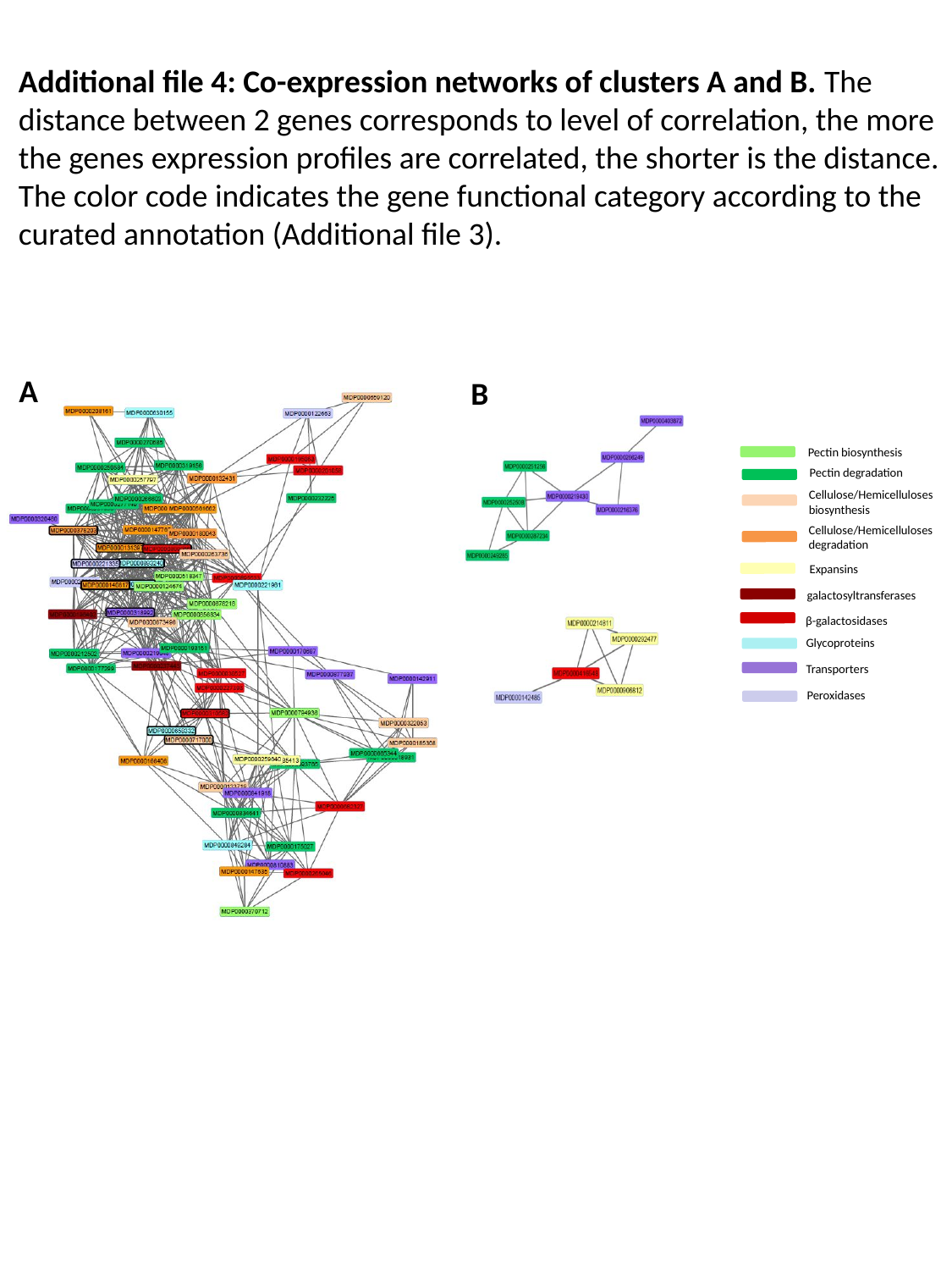

Additional file 4: Co-expression networks of clusters A and B. The distance between 2 genes corresponds to level of correlation, the more the genes expression profiles are correlated, the shorter is the distance. The color code indicates the gene functional category according to the curated annotation (Additional file 3).
A
B
Pectin biosynthesis
Pectin degradation
Cellulose/Hemicelluloses biosynthesis
Cellulose/Hemicelluloses degradation
Expansins
galactosyltransferases
β-galactosidases
Glycoproteins
Transporters
Peroxidases
