# Supplementary material for: Cell wall dynamics during apple development and storage involves hemicellulose modifications and related expressed genes
Source: BMC Plant Biol. 2016 Sep 15;16:201. doi: 10.1186/s12870-016-0887-0 (PMC5024441; doi:10.1186/s12870-016-0887-0)
Supplement: Additional file 6: — Fruit firmness evolution during cold storage. Fruit firmness was evaluated by penetrometry from harvest (H) to 2 months of cold storage (2 M). Assessment of firmness was performed on the opposite sides of each fruit in the blush and shaded regions. Force in Newtons (N) was measured at 7 mm of displacement. The bold and dash lines are respectively associated with plot PH and P12. (PPTX 81 kb) [file 12870_2016_887_MOESM6_ESM.pptx]

## Slide 1
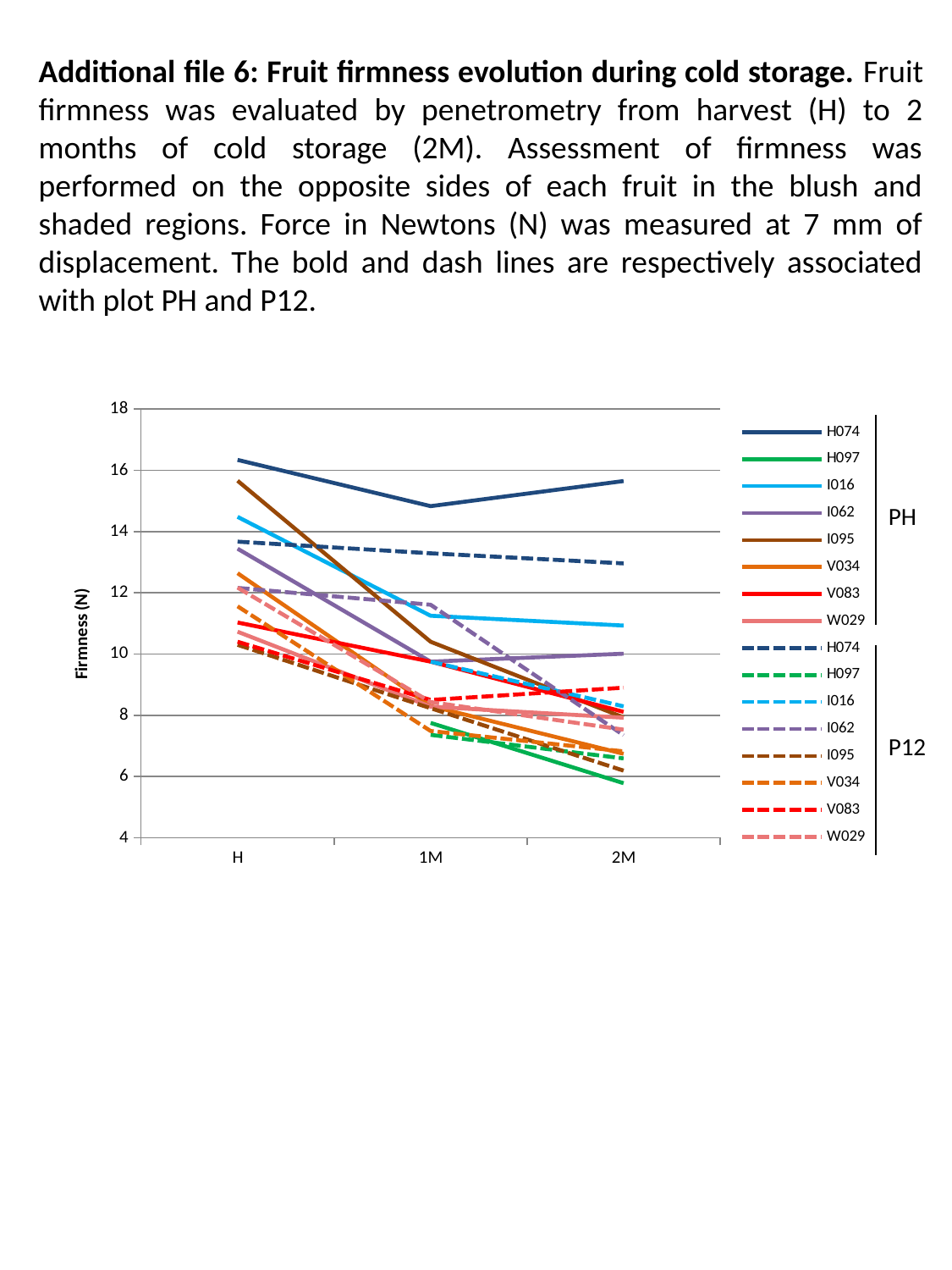

Additional file 6: Fruit firmness evolution during cold storage. Fruit firmness was evaluated by penetrometry from harvest (H) to 2 months of cold storage (2M). Assessment of firmness was performed on the opposite sides of each fruit in the blush and shaded regions. Force in Newtons (N) was measured at 7 mm of displacement. The bold and dash lines are respectively associated with plot PH and P12.
### Chart
| Category | H074 | H097 | I016 | I062 | I095 | V034 | V083 | W029 | H074 | H097 | I016 | I062 | I095 | V034 | V083 | W029 |
|---|---|---|---|---|---|---|---|---|---|---|---|---|---|---|---|---|
| H | 16.34 | None | 14.48 | 13.44 | 15.66 | 12.639999999999999 | 11.03 | 10.729999999999999 | 13.67 | None | None | 12.16 | 10.3 | 11.56 | 10.4 | 12.17 |
| 1M | 14.83 | 7.75 | 11.25 | 9.75 | 10.4 | 8.3 | 9.75 | 8.280000000000001 | 13.29 | 7.359999999999999 | 9.75 | 11.61 | 8.229999999999999 | 7.485 | 8.5 | 8.43 |
| 2M | 15.65 | 5.78 | 10.93 | 10.01 | 7.930000000000001 | 6.74 | 8.11 | 7.92 | 12.96 | 6.59 | 8.290000000000001 | 7.35 | 6.1899999999999995 | 6.819999999999999 | 8.9 | 7.53 |PH
P12
