# Supplementary material for: Cell wall dynamics during apple development and storage involves hemicellulose modifications and related expressed genes
Source: BMC Plant Biol. 2016 Sep 15;16:201. doi: 10.1186/s12870-016-0887-0 (PMC5024441; doi:10.1186/s12870-016-0887-0)
Supplement: Additional file 8: — Schema of treatments was applied on AIM. (PPTX 63 kb) [file 12870_2016_887_MOESM8_ESM.pptx]

## Slide 1
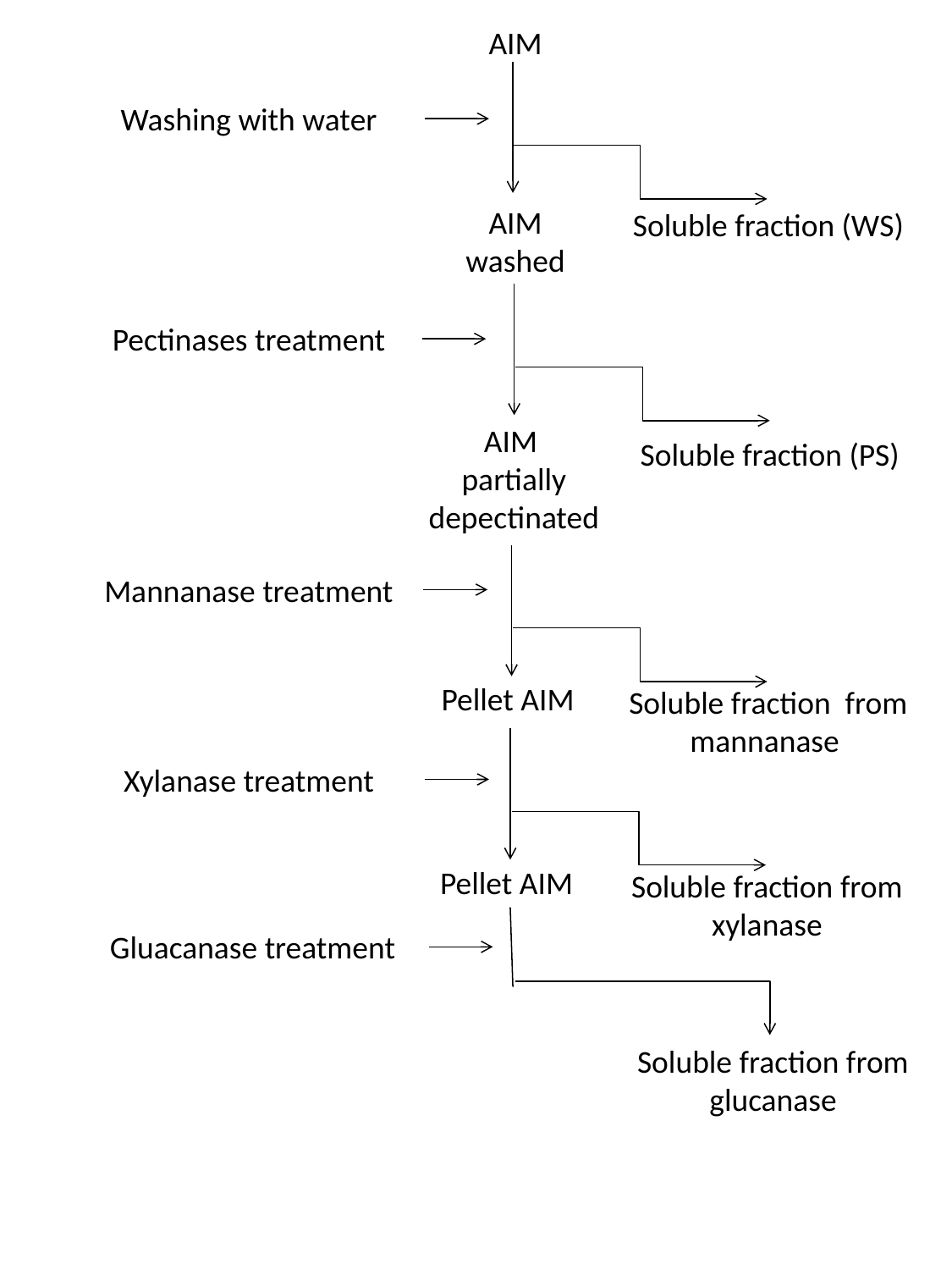

AIM
Washing with water
AIM
washed
Soluble fraction (WS)
Pectinases treatment
AIM
partially depectinated
Soluble fraction (PS)
Mannanase treatment
Pellet AIM
Soluble fraction from mannanase
Xylanase treatment
Pellet AIM
Soluble fraction from xylanase
Gluacanase treatment
Soluble fraction from glucanase
